# Anatomical Atlas and Three-Dimensional Musculoskeletal Model of the Flight Apparatus in the Southern Screamer (Anseriformes: *Chauna torquata*)

**DOI:** 10.1093/iob/obag038

**Published:** 2026-07-14

**Authors:** Klara Widrig, Aiden Starry, Helen F James

**Affiliations:** Department of Vertebrate Zoology, Smithsonian National Museum of Natural History, Washington, DC 20560, USA; Department of Vertebrate Zoology, Smithsonian National Museum of Natural History, Washington, DC 20560, USA; Department of Biology, Union University, Jackson, TN 38305, USA; Department of Vertebrate Zoology, Smithsonian National Museum of Natural History, Washington, DC 20560, USA

## Abstract

The three bird species within the family Anhimidae (screamers) are of great interest to the big picture of avian evolution due to both their phylogenetic position and their unusual combination of features. As screamers are early diverging members of Anseriformes (waterfowl), which itself is an early diverging clade within the extant bird radiation, their anatomy may help shed light on that of the common ancestor of both Anseriformes and crown-group birds as a whole. To reveal new information about the wing anatomy of anhimids, we generated a three-dimensional musculoskeletal model of the flight apparatus of the extant Southern Screamer (*Chauna torquata*) using diffusible iodine-based contrast-enhanced computed tomography (diceCT). We found that the wing muscle anatomy of *Chauna* reflects the soaring flight style employed by anhimids, as opposed to the rapid continuous wingbeats used by most other anseriforms. In *Chauna*, this manifests as a more distal insertion of the pectoralis muscle on the humerus to facilitate slower, stronger wingbeats, and a reduction in the length of antebrachial muscle origins and insertions that serves to reduce the distal inertia of the wing. Our three-dimensional dataset represents a typical musculoskeletal system of a bird with strong flight capabilities. This, combined with our observation that most flight-related muscles in *Chauna* leave recognizable osteological correlates associated with their attachments to the skeleton, means that this work can help form a basis for future comparative studies of the avian musculoskeletal system, with implications for reconstructing the flight apparatus of fossil birds.

## Introduction

**Fig. 1:**
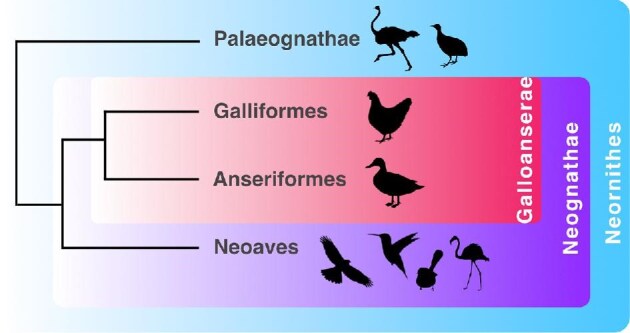
Phylogenetic tree showing relationships between avian higher level clades according to Stiller et al. (2024). *Chauna torquata*, the subject of this study, falls within Anseriformes (ducks and relatives). Silhouettes are from PhyloPic and are in the public domain.

Waterfowl (Anseriformes) and landfowl (Galliformes) together comprise the superorder Galloanserae. While representing just 4% of extant avian species diversity (Billerman et al. 2020), galloanserans represent the largest group of birds alive today in terms of overall biomass due to their being kept as livestock (Bar-On et al. 2018). This important clade has ancient origins, with the earliest galloanseran fossil exemplars dating back to the latest Cretaceous (Clarke et al. 2005; Field et al. 2020; Torres et al. 2025). The earliest evolutionary split within crown group birds (Neornithes) is between Palaeognathae (ostriches and kin) and Neognathae (which contains all other extant birds) (Hackett et al. 2008; Haddrath and Baker 2012; Jarvis et al. 2014; Claramunt and Cracraft 2015; Prum et al. 2015; Reddy et al. 2017; Kuhl et al. 2020; Stiller et al. 2024)(Fig. 1). This divergence occurred during the late Cretaceous, approximately 93 million years ago according to the latest molecular frameworks (Stiller et al. 2024). The evolutionary divergence between Galloanserae and Neoaves is the deepest split within Neognathae, having occurred approximately 88 million years ago (Stiller et al. 2024). As early diverging neognaths, galloanserans hold the potential to reveal information pertaining to the early evolution of crown group birds. Growing evidence indicates that galloanserans may have retained neornithine plesiomorphies such as the plesiomorphic palatal structure (Benito et al. 2022; Crane et al. 2025), and their phylogenetic position makes them a key group for the use of the extant phylogenetic bracket to reconstruct features of the common ancestor of all living birds (Witmer 1995).

**Fig. 2:**
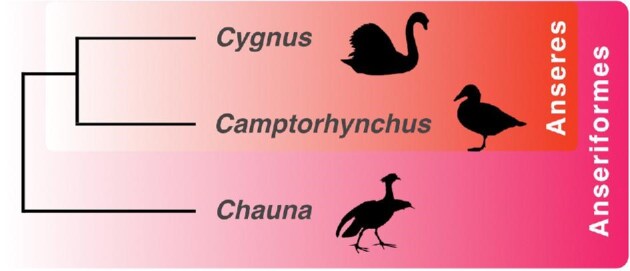
Phylogenetic tree displaying the relationships between *Chauna, Cygnus*, and *Camptorhynchus* according to the framework of Stiller et al. (2024). Silhouettes are from PhyloPic and are in the public domain.

The three species of screamer that inhabit the wetlands of South America are unique in the avian world for a multitude of reasons. These strange birds are the only extant members of the family Anhimidae, an early diverging lineage of Anseriformes that form the sister clade to the remainder of this group (Fig. 2). They certainly represent an evolutionary oddity within Anseriformes, the remainder of which is made up of the more familiar waterfowl, the ducks, geese, and swans. Screamers are large birds, typically weighing in the range of three to five kilograms (Winkler et al. 2020). Like many other anseriforms, screamers are herbivorous, and typically graze on aquatic vegetation in their wetland habitats (Winkler et al. 2020). However, unlike other anseriforms which typically possess spatulate “duck-like” bills, anhimids possess small, “landfowl-like” bills, and their large feet lack webbing. Flight in most anseriforms is characterized by rapid continuous wingbeats, whereas screamers employ their broad wings to soar for hours at a time (Hudson 1923; Brady 2020; Winkler et al. 2020). Screamers are the only extant birds which lack uncinate processes on their ribs (Winkler et al. 2020), which serve as muscle attachment sites that act as levers to increase the mechanical advantage of M. appendicostales during inspiration (Tickle et al. 2007). They exhibit a high degree of postcranial skeletal pneumatization (Burton et al. 2023), with pneumatization of limb bones extending all the way to the ends of the digits (DeMay 1940). Screamers also exhibit extensive subcutaneous pneumatization, and the internal air sac system is especially extensive (DeMay 1940). Other noteworthy features are the two large wing spurs, one on the alular metacarpal and the other on the distal carpometacarpus, that occur in both sexes, and are utilized in disputes over mates and territory (Winkler et al. 2020). Despite these differences, screamers have long been recognized as members of Anseriformes i.e., (Parker 1863; Huxley 1867; Shufeldt 1901; Pycraft 1910; Wetmore 1929; Livezey 1997; Livezey and Zusi 2007), a categorization that has received robust support from molecular phylogenetic studies (Hackett et al. 2008; Prum et al. 2015; Kimball et al. 2019; Feng et al. 2020; Stiller et al. 2024).

Some researchers posit that, with the exception of their extreme postcranial skeletal pneumaticity, screamer morphology may be closer to the ancestral anseriform condition than other members of the group. This is especially notable given their similarity to Presbyornithidae, an extinct group now believed to represent stem Anseriformes (De Pietri et al. 2016). A fossil displaying a mosaic of duck-like and screamer-like characteristics *Anachronornis anhimops* from the late Paleocene of North America was interpreted by Houde et al. (2023) as stemward of the Anhimidae-Anseres divergence and placed in a new family, Anachronornithidae. *Danielsavis nazensis* from the early Eocene London Clay of southeast England was also referred to this family, which is notable for its lack of postcranial skeletal pneumatization (Houde et al. 2023).

*Chaunoides antiquus* from the late Oligocene or early Miocene of Brazil is clearly recognizable as a screamer but not as pneumatized as extant species, and was slightly smaller and more gracile (Alvarenga 1999). A quadrate from the earliest Eocene Tingmarra fauna of Australia may represent an earlier occurrence of Anhimidae, suggesting that screamers once had a much greater geographic range (Elzanowski and Boles 2012).

Here, we focus on the screamer wing apparatus and its potential to reveal the ancestral state of crown bird flight musculature as part of an extant phylogenetic bracket. As a group, anseriforms may be more suitable for phylogenetic bracketing of the ancestral crown bird because the burst flight employed by their sister group (Galliformes) is highly derived, leading to a distinct suite of musculoskeletal characters such as elongated sterna and shortened wings (Lowi-Merri et al. 2021; Widrig et al. 2023; Widrig et al. 2025). Screamers represent an outlier from the remaining anseriforms in terms of flight style, providing an opportunity to compare the flight musculature of members of the same order that do not utilize soaring flight. Additionally, we used this project as an opportunity to test the assumption that avian antebrachial muscles leave few correlates of their origins and insertions on the skeleton (McGowan 1982), which has major implications for efforts to reconstruct the flight musculature of extinct birds.

This work expands upon the available literature regarding the flight apparatus of early-diverging Neornithine clades. As the only extant volant palaeognaths, tinamous have had their flight musculature well characterized (Hudson et al. 1972; Suzuki et al. 2014; Widrig et al. 2023), and Galliformes are similarly well studied (Hudson and Lanzillotti 1964; Harvey et al. 1969; Yasuda 2004). Publications addressing the wing myology of most higher level avian clades are available, including but not limited to: Mirandornithes (Vanden Berge 1970), Columbimorphae (George and Berger 1966), Otidimorphae (Berger 1953; Berger 1954; Berger 1960), Gruiformes (Fisher and Goodman 1955; Berger 1956b; McGowan 1986), Charadriiformes (Hudson et al. 1969; Watanabe et al. 2021), Aequornithes (Vanden Berge 1970; McKitrick 1991; Watanabe et al. 2021), Accipitriformes (Fisher 1946), Falconiformes (Berger 1956a; Meyers 1992; Meyers 1996), Psittaciformes (Razmadze et al. 2018), and Passeriformes (Hudson and Lanzillotti 1955; Berger 1956c; George and Berger 1966; Raikow 1977; Raikow 1978; McKitrick 1985; Raikow 1985). Despite being widely available through commercial farming and game hunting, surprisingly little has been published on the appendicular myology of Anseriformes, a fact remarked on by authors nearly half a century ago (Zusi and Bentz 1978). The only English-language published works on the anseriform wing apparatus are partial descriptions of that of the flightless Auckland Islands Teal *Anas aucklandica* (Livezey 1990) and the Whooper Swan *Cygnus cygnus* (Matsuoka and Hasegawa 2007), and a description of the antebrachium of the extinct Labrador Duck *Camptorhynchus labradorius* (Zusi and Bentz 1978) (Fig. 2). Some additional information can be gleaned from myological character states in Livezey and Zusi (2006) coded for *Chauna*, *Anseranas*, *Anser*, and *Anas*.

In this publication, we image and describe the flight apparatus of a Southern Screamer (*Chauna torquata*) using diffusible iodine-based contrast-enhanced computed tomography (diceCT), with additional information added from traditional dissection of the same specimen and several *Chauna* specimens in the Smithsonian National Museum of Natural History (NMNH) collections that had been previously dissected by other researchers. The diceCT method renders the soft tissues of a specimen radiopaque, enhancing soft tissue visibility in computed tomography (CT) scans such that the specimen can be digitally dissected (Gignac and Kley 2014; Gignac et al. 2016). In recent years this has become a well-established visualization tool for comparative anatomists, with diceCT data available for hundreds of vertebrate taxa (Gray et al. 2025). The primary advantages touted for this method include its less destructive nature, enabling multiple examinations of the same specimen with the muscles in situ, and the ability to easily share the resulting 3D data in online repositories (Gignac and Kley 2014; Gignac et al. 2016; Widrig et al. 2023; Blackburn et al. 2024; Gray et al. 2024; Gray et al. 2025). However, because iodine-based stains have a lower affinity for tendons than muscle tissue, tendinous attachments to bone may need to be identified through traditional dissection techniques (Widrig et al. 2023).

Digital dissection has been successfully utilized to study the appendicular anatomy of a number of vertebrates, including amphibians (Leavey et al. 2024), mammals (Fahn-Lai et al. 2020; Kissane et al. 2025), squamates (Fahn-Lai et al. 2020), and birds (Bribiesca-Contreras and Sellers 2017; Sullivan et al. 2019; Widrig et al. 2023). Guides to best practices for staining and scanning testudines (Gray et al. 2024), fish (Kolmann et al. 2023), snakes (Callahan et al. 2021), and mammals (Costello et al. 2024) are available, but none yet exists for birds. Digital dissection has been used effectively on a small but growing number of avian taxa (Tahara and Larsson 2013; Lautenschlager et al. 2014; Li and Clarke 2016; Bribiesca-Contreras and Sellers 2017; Sullivan et al. 2019; Widrig et al. 2023; Schachner et al. 2024). In experiments to determine best practices for diceCT scanning fluid-preserved avian specimens, Early et al. (2020) observed that the widely used stain of Lugol’s iodine (I_2_KI) in water led to degradation of the specimen, with bones becoming demineralized and flexible, and muscle tissue becoming soft. Lugol’s iodine or elemental I_2_ in 70% EtOH rather than water were therefore recommended as alternatives to water-based stains.

As a result of this observed specimen degradation, project leaders have become hesitant to include birds in CT datasets using fluid-preserved museum specimens for fear of causing permanent damage during the staining and destaining process.

Gray et al. (2025) performed diceCT on a large sample of mammals, fish, amphibians, and reptiles and established staining protocols for each group, but did not include birds.

The bones of a Ruby-Throated Hummingbird (*Archilochus colubris*) experimentally stained by Gray et al. (2025) in a buffered water-based Lugol’s iodine solution became flexible after destaining, indicating that the results of Early et al. (2020) are not unique. It was suggested by Gray et al. (2025) that it may in fact be the destaining process that damages fluid-preserved birds.

In addition to contributing to our knowledge of avian wing morphology, the present study serves as an opportunity to test the contrast staining methods recommended by Early et al. (2020) on a larger avian specimen. Larger specimens typically take a greater amount of time to stain (Gray et al. 2025), and the majority of diceCT work has focused on smaller specimens under 1 kg, making the appropriate methods for larger specimens less clear (Costello et al. 2024). By choosing to stain and scan a screamer, our goals are to fill a crucial gap in the literature on anseriform anatomy, further investigate the effects of staining and scanning on fluid preserved birds, as well as provide general information related to staining and scanning larger specimens that will be applicable to the wider diceCT community.

## Materials and methods

### Specimen

The *Chauna torquata* specimen (USNM 666527) was obtained by the Smithsonian National Museum of Natural History (hereafter NMNH) from the Ripley Waterfowl Conservancy in Litchfield, Connecticut, USA after dying at the age of 18 years from natural causes. The specimen, a captive-born adult female, weighed 3200 grams at death and had been frozen prior to fixing and staining. Although the flight feathers had been clipped to prevent the bird from flying, we confirmed by X-ray that the bird was not pinioned (a surgical procedure which removes the carpometacarpus distal to the alula, rendering the bird permanently flightless). We recognize that artifacts may have been introduced to our dataset from this individual, which spent the duration of its life in captivity and unable to fly (Baumgart et al. 2025). Flight muscles may be atrophied in comparison with those of a wild-caught specimen, but aside from not being a reliable source for muscle masses and volumes that would be typical of a wild bird, all origins and insertions in our specimen should be the same.

**Fig. 3:**
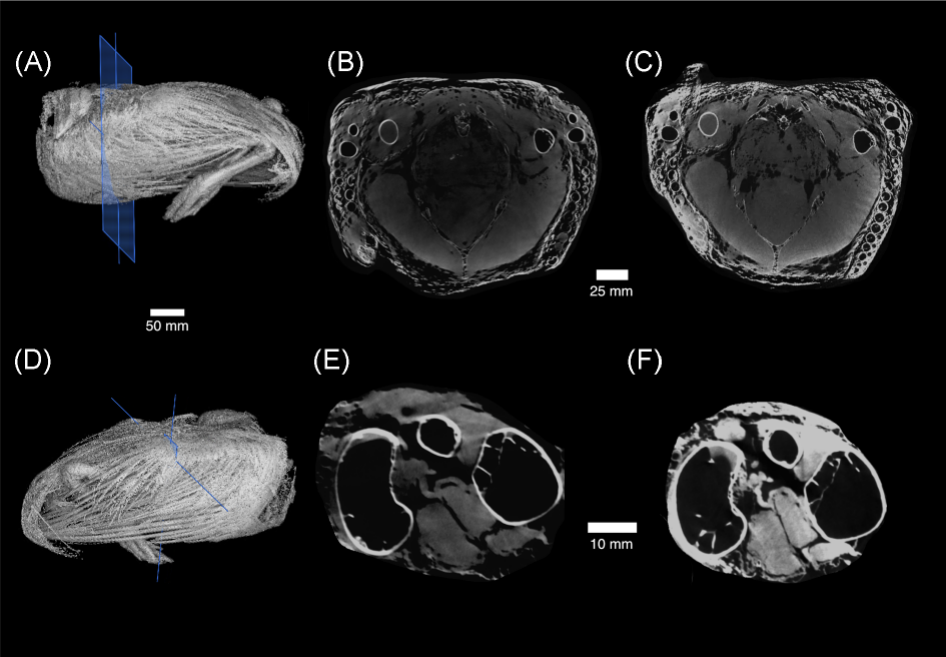
Staining progress at 42 and 64 days of staining. A: whole bird (USNM 666527) in left lateral view with blue clipping plane showing the approximate area from which cross sections B and C were taken. B: Cross section from the thorax at 42 days of staining. C: Cross section from the thorax at 64 days of staining. D. Whole bird (USNM 666527) in right lateral view with blue clipping plane showing the approximate area from which cross sections E and F were taken. E: Cross section of the elbow at 42 days of staining. F: cross section of the elbow at 64 days of staining.

Prior to fixation and staining, a tissue sample was obtained from the thigh musculature. The specimen was then injected with and soaked in formalin as a fixative before being successively transferred to 25%, 50%, and finally 70% ethanol following the protocol of Simmons (2014). Following this, the specimen was stained in elemental iodine in 70% ethanol per the methods of Early et al. (2020). A 1.25% Lugol’s iodine solution in EtOH is preferable for visualizing muscle attachments to bone as it maintains high contrast of bone relative to soft tissue as opposed to 1.25% Lugol’s iodine in H_2_O (Early et al. 2020; Crowell et al. 2025), but no information is available as to whether this applies to elemental iodine as well. Elemental iodine (I_2_) was chosen over Lugol’s iodine as Early et al. (2020) observed that Lugol’s iodine in 70% EtOH produced a grainy scan. As the specimen was extremely buoyant due to the extensive postcranial skeletal and skin pneumatization found in anhimids, a weight was attached to the specimen to ensure that it remained fully submerged throughout this process. A test scan was taken after 42 days of staining, following which the specimen was transferred to a fresh batch of iodine stain and injected with stain in the right pectoral region, brachium, antebrachium, and manus to speed diffusion. The final scan was taken after 64 total days of staining (Fig. 3). After the final scan, the specimen was moved into fresh 70% EtOH to begin the process of destaining. As no formal protocol for destaining large avian specimens exists, the solution was refreshed whenever it was observed to be discolored. This was done 48, 66, and 129 days post removal from the staining solution. When the right wing of the specimen was dissected at 196 days post removal from the staining solution, the muscles were observed to have returned to their natural coloration with no visual evidence of stain.

### Imaging

Specimen USNM 666527 was scanned on a GE phoenix v|tome|x m microCT scanner at the NMNH. Scanning parameters were: 190 kV, 250 μA, 2936 projections, voxel size 144.78 μm. Prior to scanning, the specimen was removed from the staining solution, patted dry, and placed in a heat-sealed plastic container to prevent desiccation (Callahan et al. 2021).

### 3D model construction

Each muscle and bone was digitally segmented in VG Studio Max (Volume Graphics GmbH version 2024.2) from the same scan using the “draw” tool. Each segmented region was converted into a .stl mesh file and exported into the open-source mesh processing program MeshLab (Cignoni et al. 2008). In this program, meshes were edited using the Alpha Wrap tool to remove surface artifacts from segmenting. The edited meshes were then imported into the open-source 3D modelling software Blender, where the meshes were further smoothed using the “smooth” tool in Sculpt Mode, and color was added using Sculpt Mode’s “paint hard pressure” tool. Muscle origin and insertion areas on bones were illustrated using this same tool. Final image capture for all figures in this publication was performed in Blender unless otherwise specified.

### Musculoskeletal nomenclature and description

All anatomical terminology reflects that outlined by Baumel and Witmer (1993), with English equivalents used where appropriate. Muscles were described based on the original regions of interest produced in VG Studio, with additional information from dissected specimens for muscles whose origins and insertions could not be observed in the CT dataset. A manual dissection of the manus, antebrachium, and caudal brachium was performed on USNM 666527. Two *Chauna torquata* specimens from the USNM fluid collections that had previously been dissected by other researchers were also referenced: USNM 226502, a partially dissected juvenile specimen, and USNM 508683, a heavily dissected adult that had been fully skinned. Osteological corelates of muscle attachments on bones were identified on skeletal specimens USNM 345619 and USNM 646637, which were adult birds sourced from the National Zoo.

## Results: anatomical description

### M. latissimus dorsi (Fig. 4, Fig. 5)

Pars cranialis and pars caudalis of this muscle were difficult to differentiate from one another within our CT dataset. Pars cranialis has a sheetlike origin that could be visualized on the CT scan on the neural spines of the last cervical and first two thoracic vertebrae. In USNM 226502, we observed it originating from the neural spines of the last cervical and first three thoracic vertebrae. Pars caudalis originates partly as a fleshy slip from the fifth thoracic rib, but its main caudalmost point of origin, typically on the ilium in most birds, could not be observed on the scans. In USNM 226502, we observed it originating from the neural spine of the caudalmost thoracic vertebra and the proximal dorsal ilium. The broad, thin muscle tapers to a flat sheet that passes between the bellies of M. humerotriceps and M. scapulotriceps to insert on the proximal caudal humeral shaft. Upon dissection of the scanned specimen (USNM 666527), it was revealed that the belly of pars caudalis lies ventral to that of pars cranialis, and pars caudalis grows thinner and becomes indistinguishable from pars cranialis close to the muscle’s insertion. The insertion leaves two very clear scars: one a long lateral scar, the other a smaller circular scar proximal to this lateral one that represents the shared insertion site of this muscle and the humeral anchoring tendon of M. scapulotriceps. In the CT dataset, the muscle insertion appears to span the entire area including that between the muscle scars, indicating that the scars correspond to two separate tendinous insertions with a fleshy insertion between them. We did not locate M. latissimus dorsi pars metapatagialis, but it is present in *Chauna* according to Livezey and Zusi (2006).

**Fig. 4:**
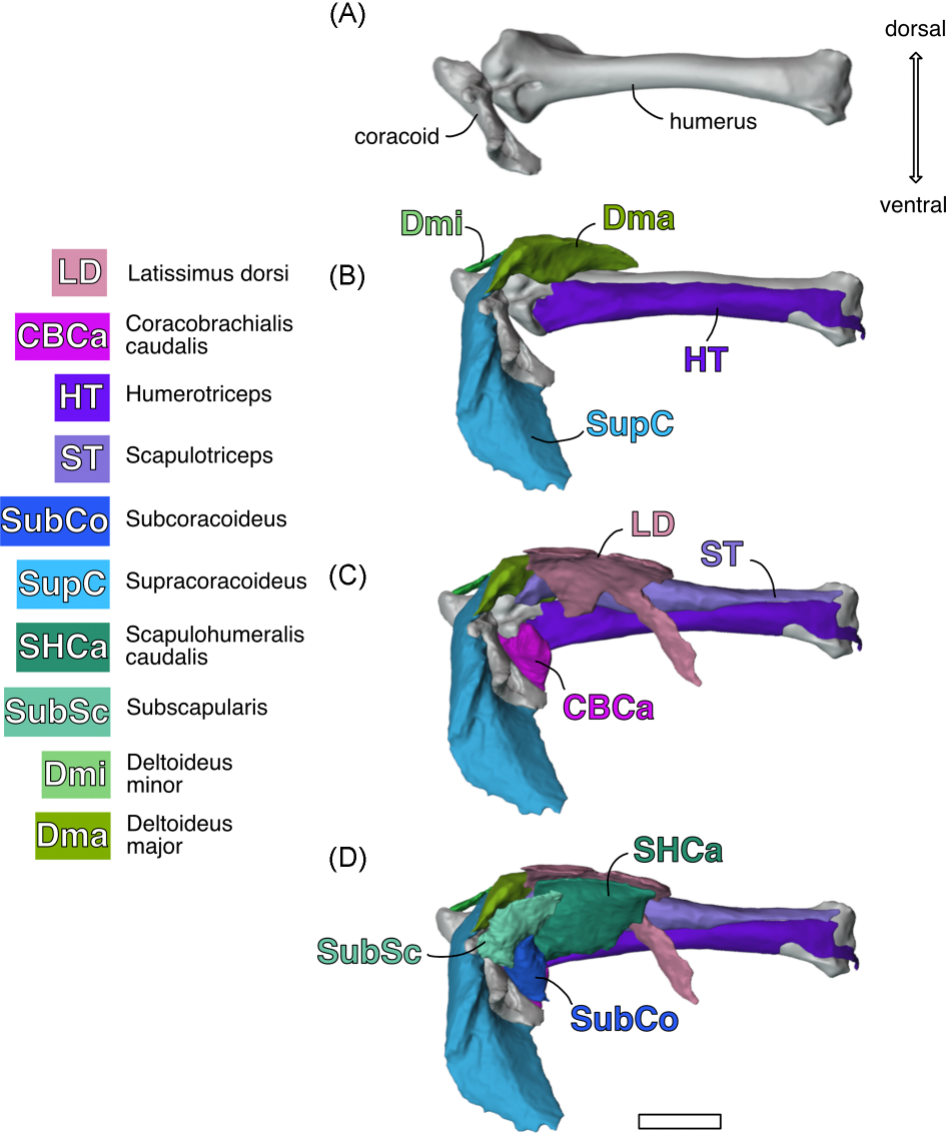
Digitally segmented osteology and myology of the coracoid and caudal humerus of *Chauna torquata*. A: right humerus and coracoid. B: same as A, with Mm. deltoideus minor, deltoideus major, supracoracoideus, and humerotriceps added. C: same as B, with Mm. coracobrachialis caudalis, latissimus dorsi, and scapulotriceps added. D: same as B, with Mm. subscapularis, subcoracoideus, and scapulohumeralis caudalis added. Scale bar = 40 mm.

**Fig. 5:**
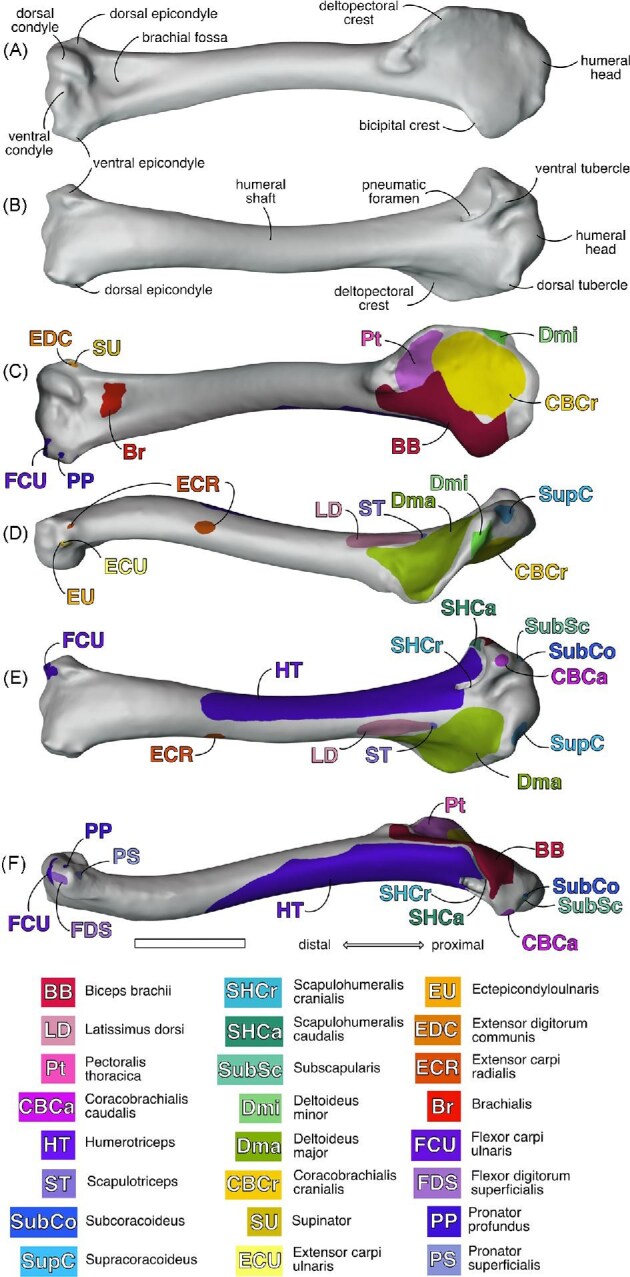
Muscle origins and insertions on the humerus. A: Right humerus in cranial view, with osteological features labelled. B: Right humerus in caudal view, with osteological features labelled. C: Same as A, with muscle origins and insertions highlighted. D: Right humerus in dorsal view, with muscle origins and insertions highlighted. E: Same as B, with muscle origins and insertions highlighted. F: Right humerus in ventral view, with muscle origins and insertions highlighted. Scale bar = 40 mm.

### M. supracoracoideus (**Figs 4**-**7**)

The origin of this muscle is fleshy, from the proximal half of the sternal keel and extending cranially over much of the ventral surface of the coracoid. It lies deep to M. pectoralis on the sternal keel. The area of origin is delineated by a clear intermuscular line on the keel, and an intermuscular line on the ventrolateral body of the coracoid separating it from the origin of M. coracobrachialis caudalis. The thick tendon of this muscle passes through the triosseal canal and inserts on the proximal face of the dorsal tubercle of the humerus.

**Fig. 6:**
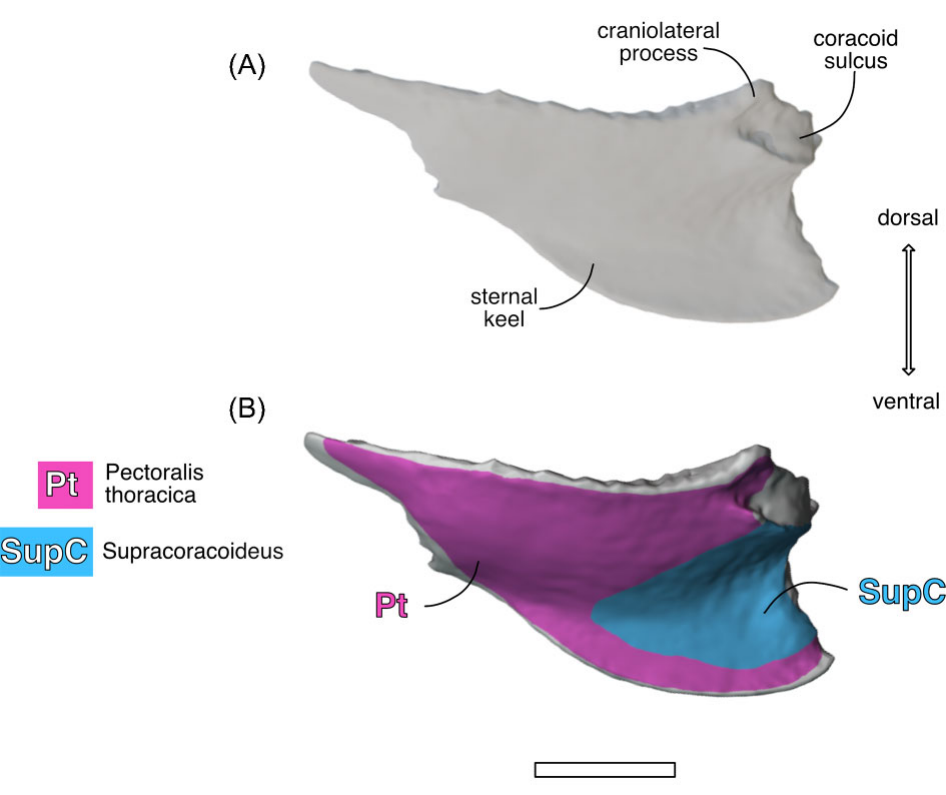
Muscle origins on the sternum. A: Sternum in right lateral view, with osteological features labelled. B: Same as A, with muscle origin areas highlighted. Scale bar = 40 mm.

**Fig. 7:**
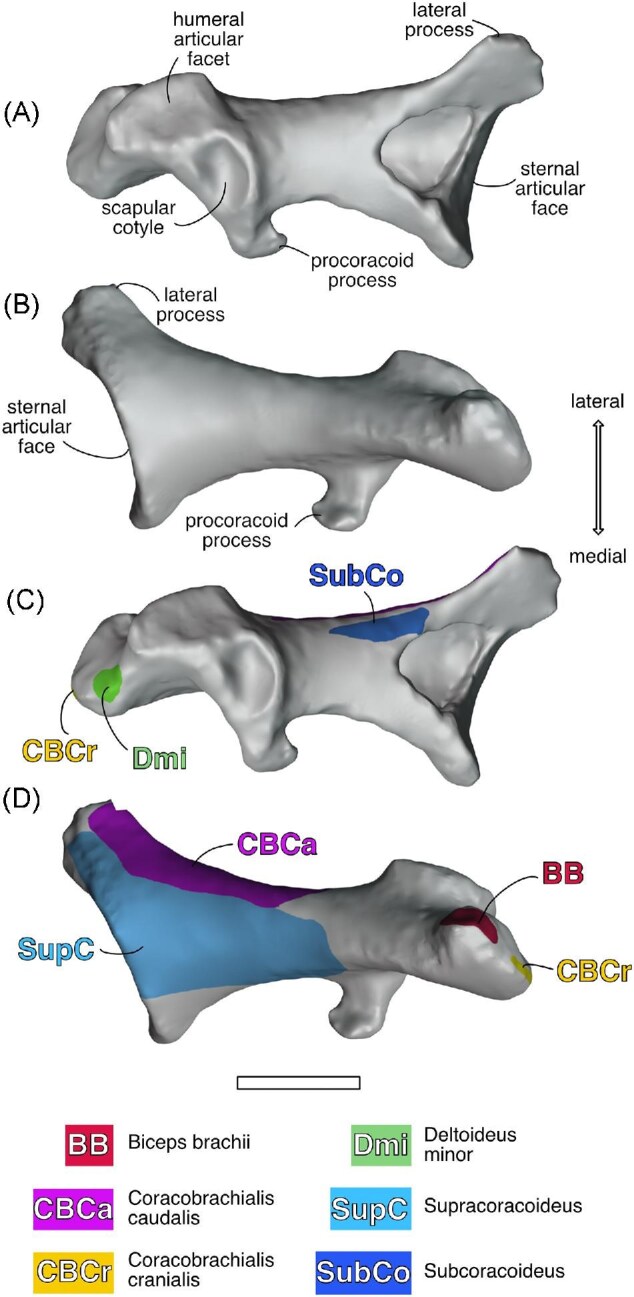
Muscle origins on the coracoid. A: Right coracoid in dorsal view, with osteological features labelled. B: Right coracoid in ventral view, with osteological features labelled. C: same as A, with muscle origin areas highlighted. D: same as B, with muscle origin areas highlighted. Scale bar = 20 mm.

### M. **pectoralis** (**Figs 5**, **6**, **8**-**10**)

This massive muscle is the largest in the body. The origin is fleshy from the entire ventral surface of the sternal body, the membrane overlying M. supracoracoideus, the posterior half of the sternal keel extending to its caudalmost point, and extending cranially onto the sternolateral arm of the furcula. Intermuscular lines on the sternal keel separate the origin area of this muscle from that of M. supracoracoideus, and its furcular origin is indicated by intermuscular lines and an area of rugose texture on the bone’s surface. Closer to its insertion, there is a broad area of contact between this muscle and M. coracobrachialis cranialis. The massive, mixed fleshy and tendinous insertion is on the distal cranial face of the deltopectoral crest of the humerus, where it leaves a circular, rugose scar.

**Fig. 8:**
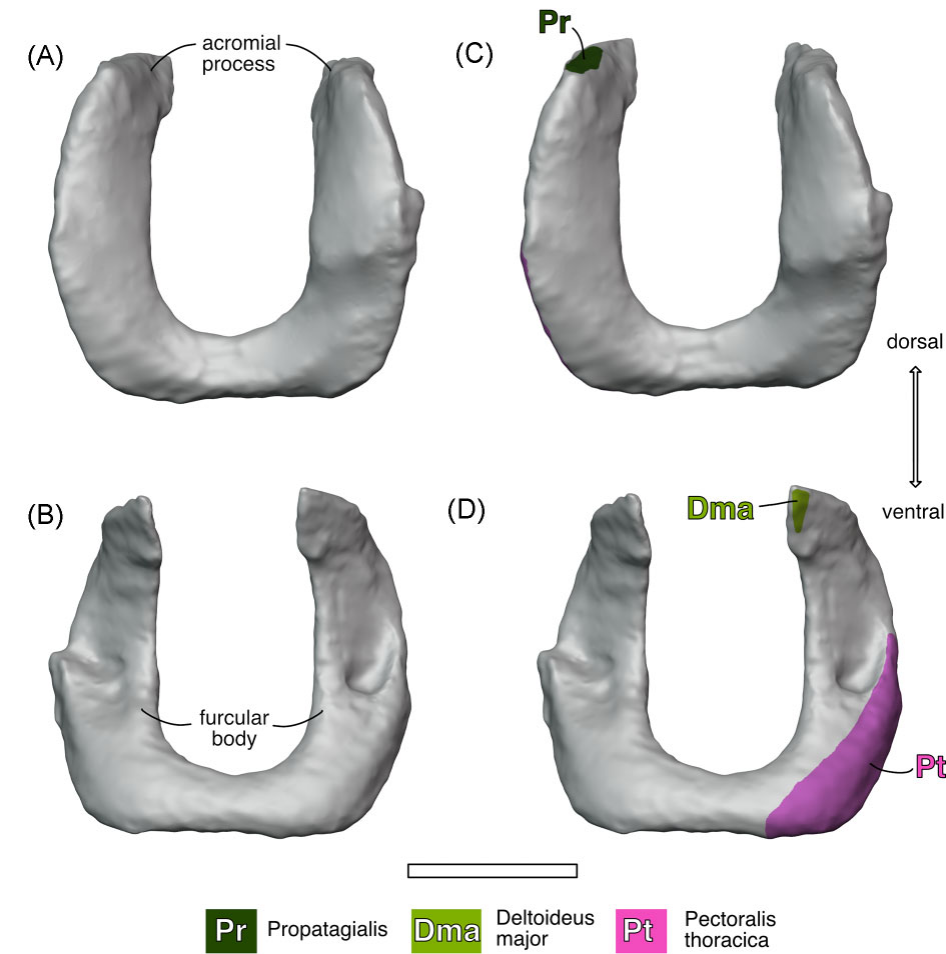
Muscle origins on the furcula. A: furcula in cranial view, with osteological features labelled. B: furcula in caudal view, with osteological features labelled. C: same as A, with muscle origins highlighted. D: same as B, with muscle origins highlighted. Scale bar = 30 mm.

**Fig. 9:**
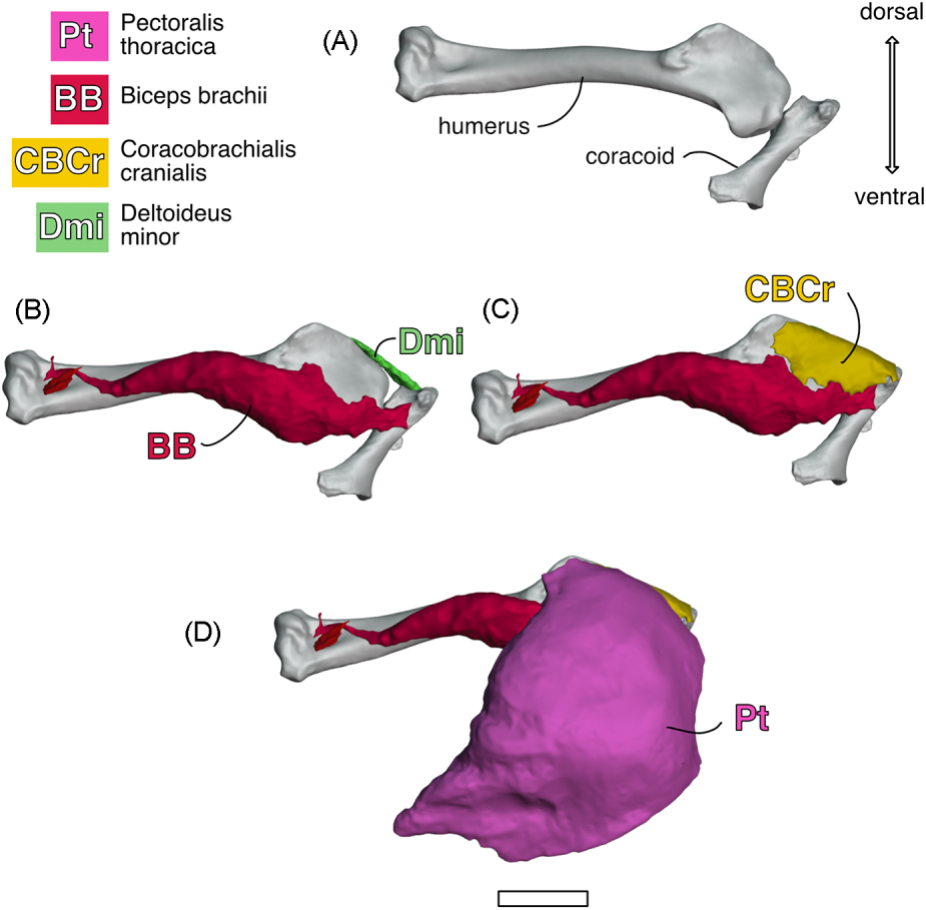
Digitally segmented osteology and myology of the coracoid and cranial humerus of *Chauna torquata*. A: right humerus and coracoid. B: same as A, with Mm. biceps brachii and deltoideus minor added. C: same as B with M. coracobrachialis cranialis added. D: same as C with M. pectoralis thoracica added. Scale bar = 40 mm.

**Fig. 10:**
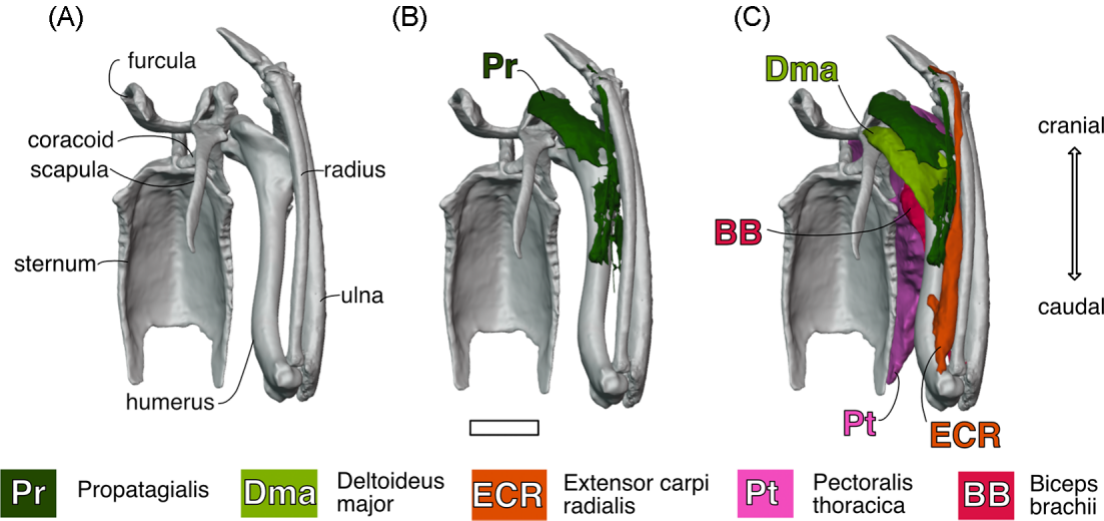
Digitally segmented osteology and myology of the propatagialis complex of *Chauna torquata*. A: Sternum, furcula, right coracoid, scapula, humerus, radius, and ulna in dorsal view. B: Same as A, with M. propatagialis added. C: Same as B, with Mm. deltoideus major, extensor carpi radialis, pectoralis thoracica, and biceps brachii added. Scale bar = 40 mm.

### M. **scapulohumeralis cranialis** (**Figs 5**, **11**)

This muscle was not visible in our CT dataset, and it was listed as vestigial or absent by Livezey and Zusi (2006). Upon dissection of the stained specimen, this muscle was found to be minute and threadlike, with a diameter of approximately 1mm. The origin was found to be directly caudal to that of M. scapulotriceps on the scapula, and the insertion is on the crest extending distally from the ventral tubercle, dorsal to the pneumatic foramen. There are no osteological correlates associated with this muscle.

**Fig. 11:**
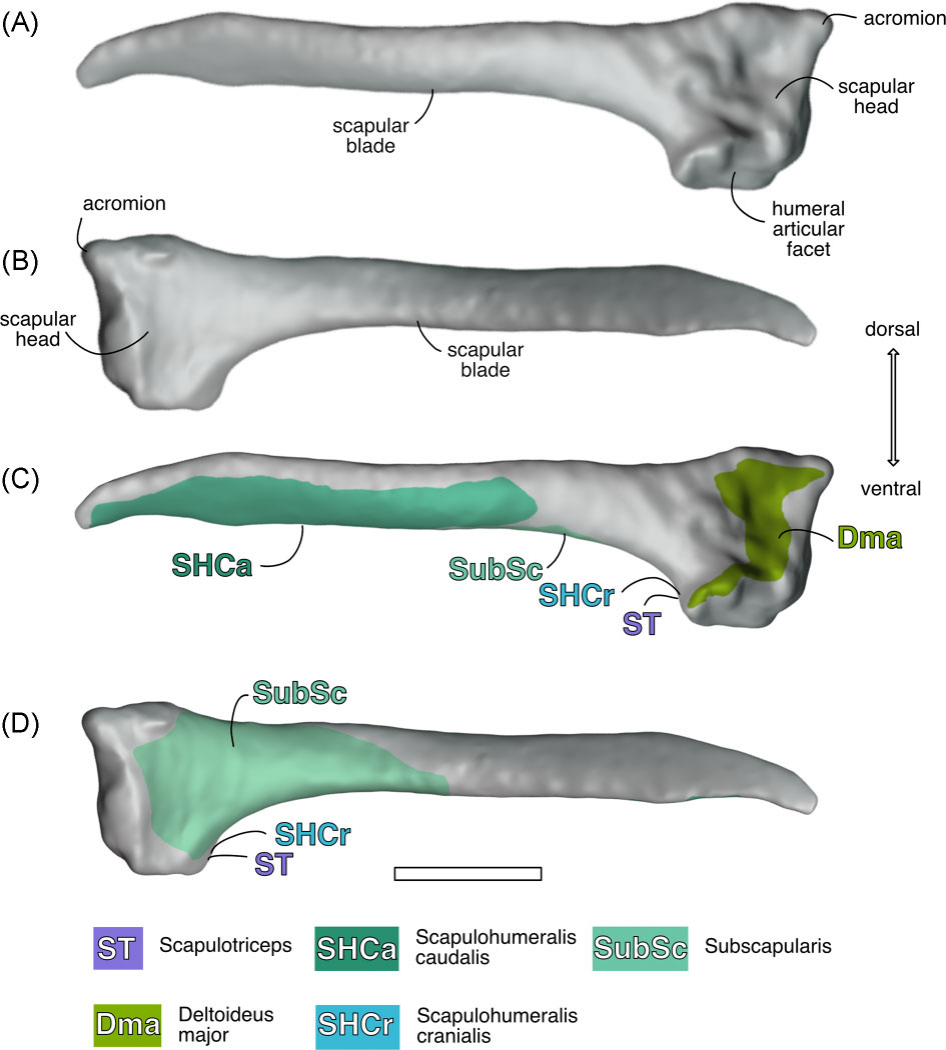
Muscle origins on the scapula of *Chauna torquata*. A: Right scapula in dorsolateral view, with osteological features labelled. B: Right scapula in ventromedial view, with osteological features labelled. C: Same as A, with muscle origin areas highlighted. D: Same as B, with muscle origin areas highlighted. Scale bar = 20 mm.

### M. **scapulohumeralis caudalis** (**Figs 4**, **5**, **11**)

The origin is fleshy and extends down the distal three quarters of the lateral scapular blade. The origin is marked by a rugose area on the lateral scapula, bounded by intermuscular lines and a proximal rugose tubercle. It extends ventrally as a broad sheet of muscle before narrowing to a tendon, which inserts as a wide sheet on the crus ventrale of the humerus and leaves an oval rugose scar.

### M. subscapularis (Figs 4, 5, 11)

This muscle has a wide, fleshy origin on the ventral and medial proximal scapula, extending from the scapular head down approximately one third of the scapular blade. Faint intermuscular lines associated with this muscle’s origin are visible on the ventrolateral scapula, but none exist on the medial scapula. The insertion is located on the proximal face of the ventral tubercle of the humerus via a shared tendon with M. subcoracoideus, which together leave a circular rugose area on the bone at the site of insertion.

### M. **subcoracoideus** (Figs 4, 5, 7)

The origin is a thin strip on the lateral side of the sternal end of the dorsal face of the coracoid, extending from the lateral process to approximately halfway up the coracoid shaft. Faint intermuscular lines are present on the dorsal shaft of this bone. This muscle inserts on the humerus via a shared tendon with M. subscapularis.

### M. **deltoideus pars major** (Figs 4, 5, 8, 10, 11)

This large muscle has a fleshy origin that covers much of the lateral head of the scapula and extends onto the ventrolateral aspect of the furcular head. The fleshy insertion is broad, covering nearly all of the caudal surface of the deltopectoral crest and extending distally onto the humeral shaft. Despite its relatively large size, there are few osteological correlates associated with this muscle. A smooth area on the otherwise rugose ventral furcular head may be associated with this muscle’s origin, but no marks are present on the scapula. Some extremely faint intermuscular lines are present in some osteological specimens on the caudal deltopectoral crest.

### M. deltoideus pars minor (**Figs 4**, **5**, **7**, **9**)

As implied by its name, this muscle is much smaller than pars major. Its origin lies on the medial head of the coracoid, abutting the origin of M. coracobrachialis cranialis, with no associated osteological correlates. It is closely associated with M. coracobrachialis cranialis throughout its length. Its insertion wraps around the dorsal tubercle of the humerus on its cranial side, distal to the insertion of M. supracoracoideus. Some faint intermuscular lines just distal to the dorsal tubercle appear to be associated with this muscle.

### M. **propatagialis** (**Figs 8****,**  **10****,**  **12**)

This muscle is unique to the avian lineage, and its developmental origin is tied to muscle progenitor cells originating from the developing Mm. deltoideus major, pectoralis, biceps brachii, and extensor carpi radialis (Uno and Hirasawa 2023; Uno and Hirasawa 2025). The origin point of this muscle is located on the dorsal apex of the furcular head, which corresponds to a rugose area on the bone. It extends superficial to the muscles of the proximal humerus, overlying Mm. deltoideus major, deltoideus minor, and coracobrachialis cranialis as a broad, thin sheet. Fibers from the sheath covering the main body of M. pectoralis wrap over the deltopectoral crest to join the body of M. propatagialis, representing the so-called “M. pectoralis propatagialis” of some authors. The elongated, tendinous portion of M. propatagialis that inserts on the carpometacarpus branches off from the broader, sheetlike portion of the muscle just distal to the deltopectoral crest. The so-called “biceps slip” connects the sheetlike portion of the muscle to M. biceps brachii near the approximate midpoint of the humeral shaft. The tendinous portion of M. propatagialis receives additional fibers from the proximal fleshy belly of M. extensor carpi radialis. It extends down the leading edge of the wing encased by a tube of thickened dermis, and inserts immediately ventral to M. extensor carpi radialis on the proximal portion of the extensor process, which in anhimids is elongated into a large spur. An elongate scar corresponds to the insertions of this muscle and those of Mm. extensor carpi radialis and extensor longus alulae.

**Fig. 12:**
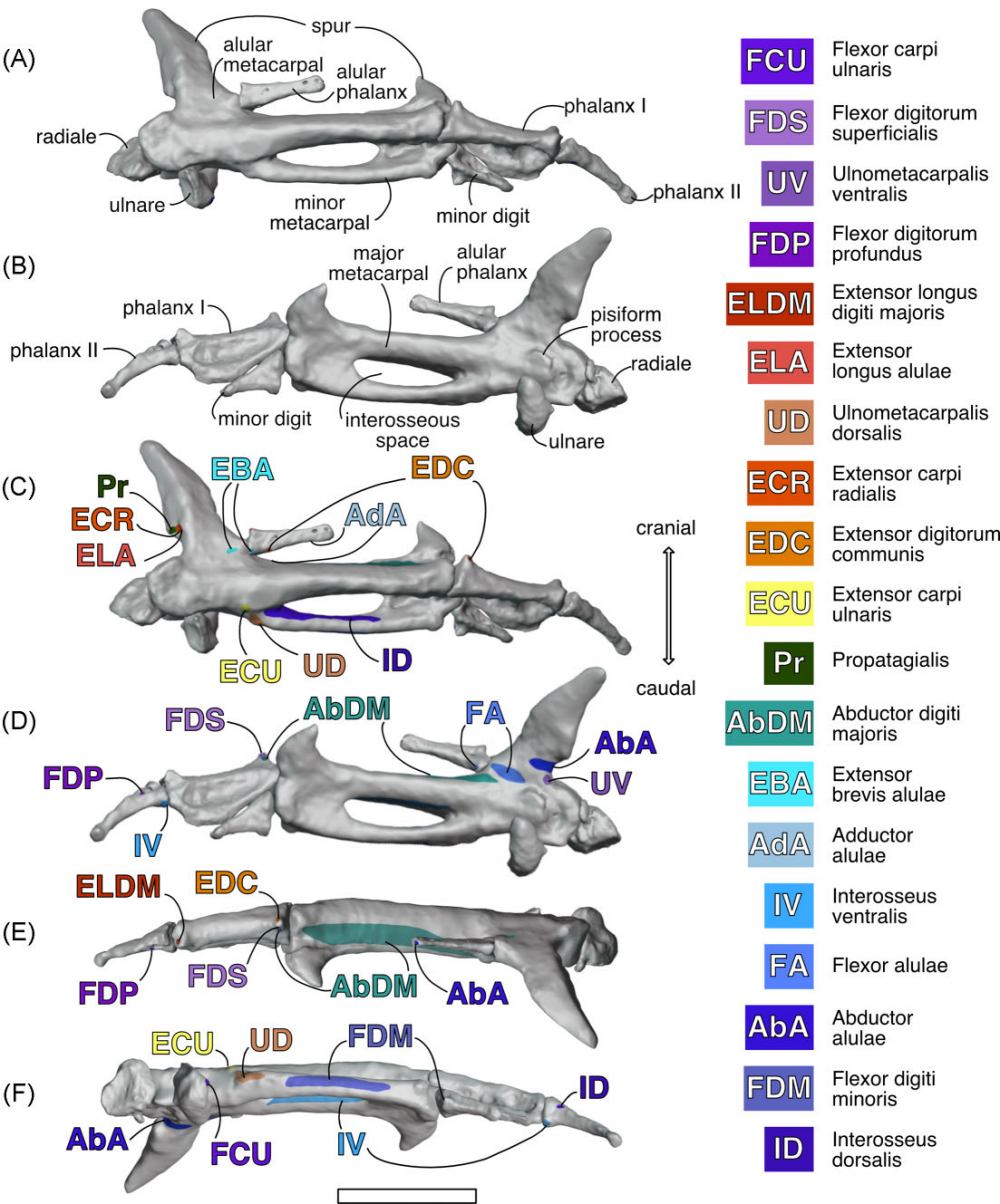
Muscle origins and insertions on the manus and carpal elements of *Chauna torquata*. A: Right manus in dorsal view, with osteological features labelled. B: Right manus in ventral view, with osteological features labelled. C: Same as A, with muscle origins and insertions highlighted. D: Same as B, with muscle origins and insertions highlighted. E: Right manus in cranial view, with muscle origins and insertions highlighted. F: Right manus in caudal view, with muscle origins and insertions highlighted. Scale bar = 40 mm.

### M. **coracobrachialis cranialis** (**Figs 5**, **7**, **9**)

This muscle is well-developed in *Chauna*. The origin is located on the dorsal head of the coracoid, covering the acrocoracohumeral ligament and extending onto a small strip of the coracoid itself. There is no osteological correlate of the origin of this muscle. The extensive fleshy insertion covers the coracobrachial impression on the proximal cranial humerus and extends onto the proximal half of the deltopectoral crest. Unusually among birds, the insertion leaves faint intermuscular lines on the deltopectoral crest, but the coracobrachial impression is not clearly defined.

### M. **coracobrachialis caudalis** (**Figs 4**, **5**, **7**)

The origin of this muscle extends from the ventrolateral sternal end of the coracoid up onto the lateral coracoid shaft, in close association with M. subcoracoideus. An intermuscular line separates it from the coracoidal origin of M. supracoracoideus. The insertion is by a diffuse tendon covering the apex of the ventral tubercle of the humerus, which leaves a large, ovoid impression with a rugose texture.

### M. **scapulotriceps** (**Figs 4**, **11**, **13**)

The tendinous origin of M. scapulotriceps is on the proximal scapula immediately caudal to the glenoid, where a circular rugose tubercle can be found. The muscle immediately expands into a large, fleshy belly as it extends down the length of the dorsocaudal humeral shaft. It is anchored to the proximal portion of the caudal humeral shaft via a stout tendon that also receives fibers from M. latissimus dorsi. This anchoring tendon leaves a prominent circular scar just proximal to the linear scar associated with M. latissimus dorsi. The belly of M. scapulotriceps is in contact with the dorsal portion of M. humerotriceps over much of its length. Distally, it narrows to a tendon and cannot be observed in our CT data distal to the dorsal condyle of the humerus due to the presence of a thick sheath of connective tissue. It inserts in common with M. humerotriceps on the olecranon of the ulna, leaving a faint scar.

**Fig. 13:**
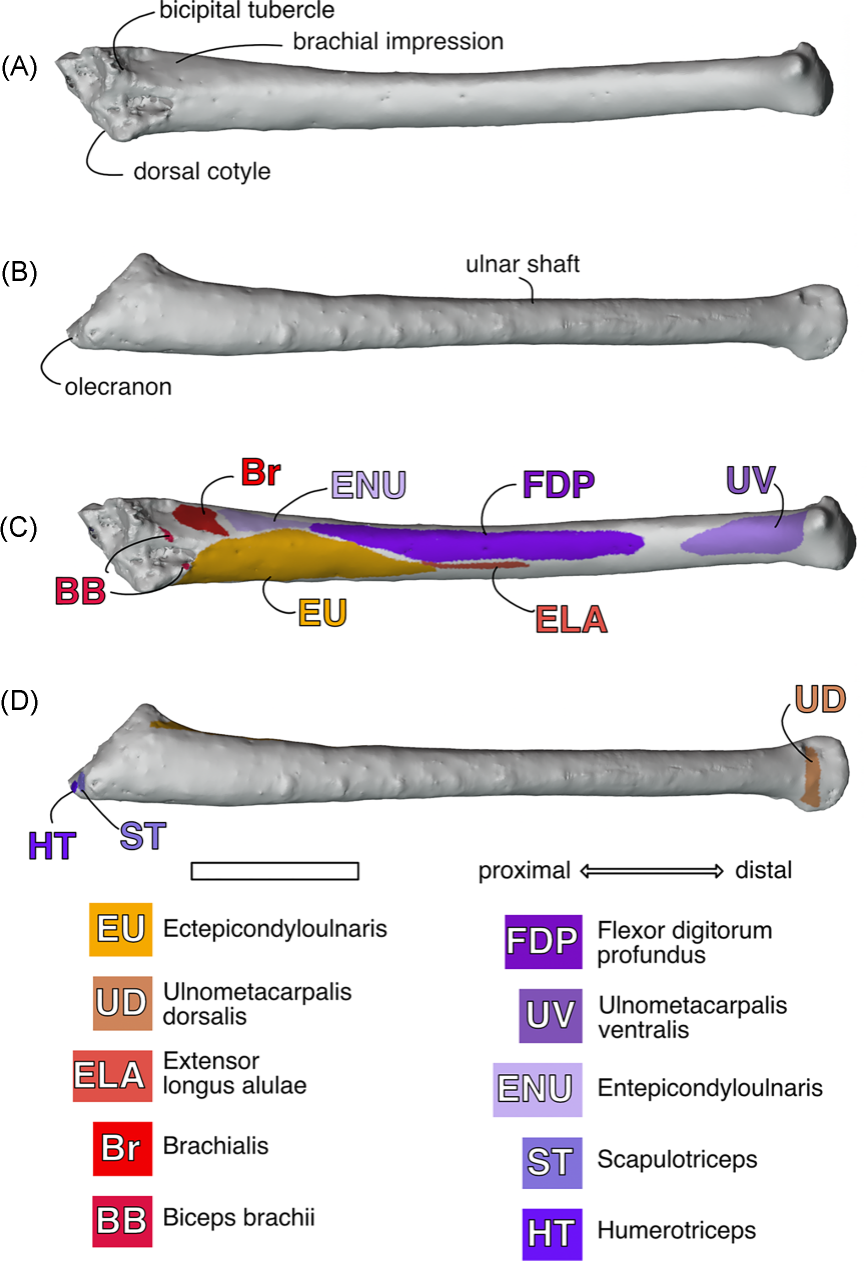
Muscle origins and insertions on the ulna of *Chauna torquata*. A: Right ulna in ventral view, with osteological features labelled. B: Right ulna in dorsocaudal view, with osteological features labelled. C: Same as A, with muscle origins and insertions highlighted. D: Same as B, with muscle origins and insertions highlighted. Scale bar = 40 mm.

### M. **humerotriceps** (**Figs 4**, **5**, **13**)

This muscle covers much of the caudal surface of the humerus. The origin is fleshy on the caudal humeral head, extending into the pneumatic fossa. The origin is slightly bifurcated by the crus dorsale. In many birds, the proximal area of origin of this muscle is clearly marked by intermuscular lines. We observed these intermuscular lines in a variety of bird genera within the collections of the NMNH, but none could be found for *Chauna*. The belly of the muscle contacts the humeral shaft for nearly all of its length. It becomes tendinous distally, and inserts on the olecranon of the ulna.

### M. **biceps brachii** (**Figs 5**, **7**, **9**, **10**, **13**, **14**)

The origin is on the lateral head of the coracoid via a sheetlike tendon, ventral to the origin of M. coracobrachialis cranialis, and extends onto the bicipital crest and dorsally up the ventral portion of the cranial face of the proximal humerus. A clear facet on the lateral coracoid head is associated with this muscle’s origin. As it approaches the distal head of the humerus, the muscle becomes tendinous. This tendon then splits in two, inserting onto the bicipital tubercles of the radius and ulna.

**Fig. 14:**
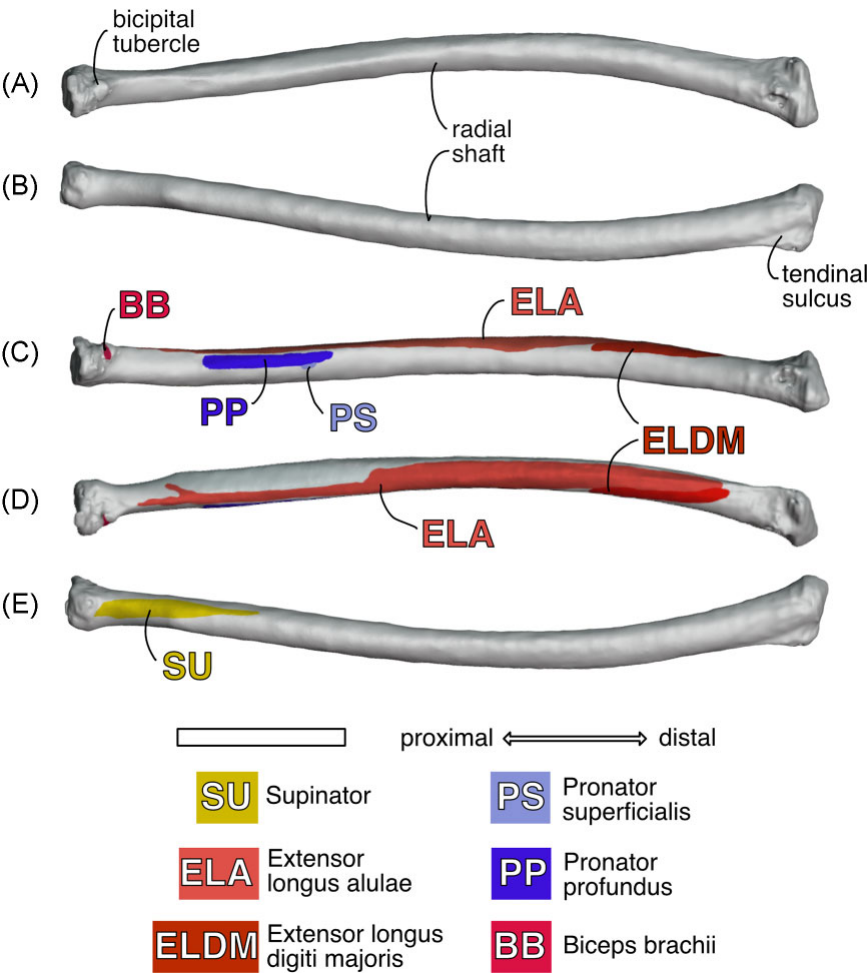
Muscle origins and insertions on the radius of *Chauna torquata*. A: Right radius in caudal view, with osteological features labelled. B: Right radius in cranial view, with osteological features labelled. C: Same as A, with muscle origins and insertions highlighted. D: Right radius in ventrocaudal view, with muscle origins highlighted. E: Same as B, with muscle insertion highlighted. Scale bar = 40 mm.

### M. **brachialis** (**Figs 5**, **13**, **15**)

The origin of this muscle is in the deeply excavated brachial fossa of the distal cranial humerus. It stretches across the elbow joint to insert on the brachial impression of the proximal ventral ulna, deep to the insertion of M. entepicondyloulnaris.

**Fig. 15:**
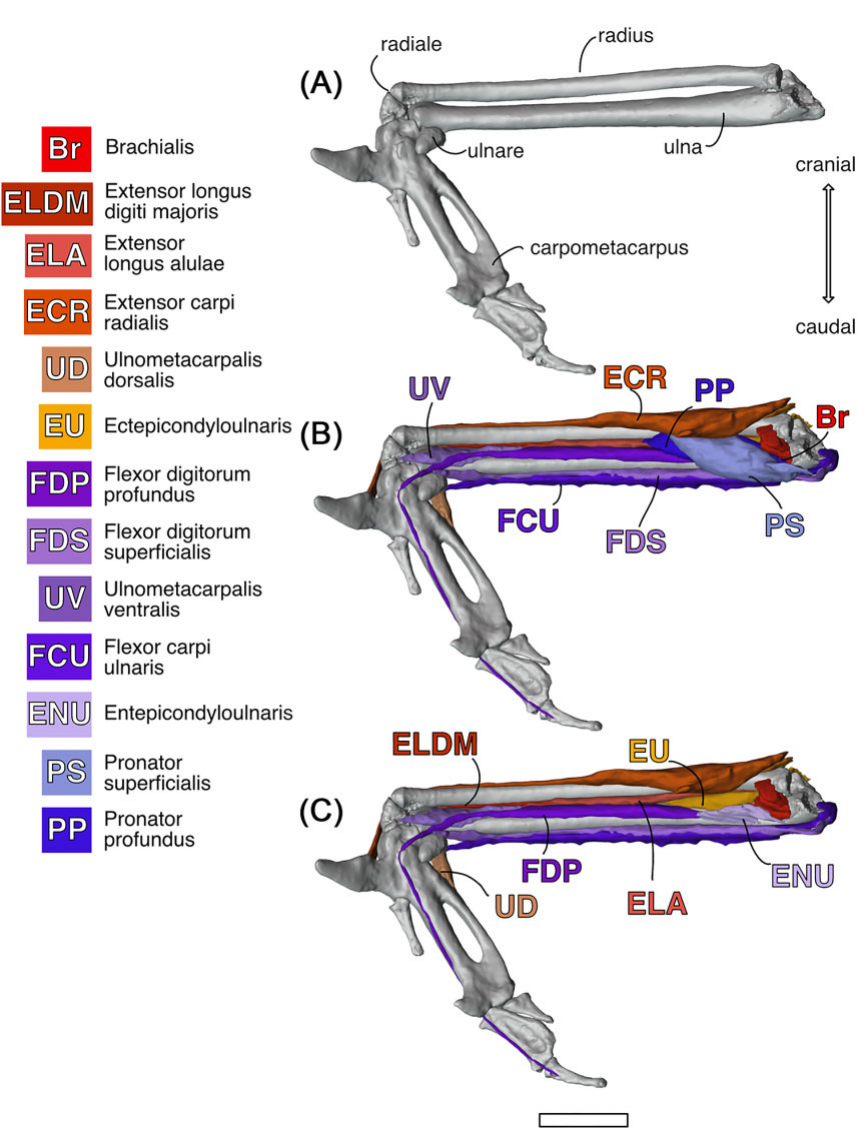
Digitally segmented ventral forearm of *Chauna torquata*. A. Right radius, ulna, radiale, ulnare, carpometacarpus, and phalanges. B. same osteology as A, with right Mm. brachialis, pronator superficialis, pronator profundus, extensor carpi radialis, flexor digitorum superficialis, flexor carpi ulnaris, and ulnometacarpalis ventralis. C. same osteology as A, with Mm. pronator profundus and pronator superficialis removed to display the deeper muscles of the forearm: right Mm. entepicondyloulnaris, ectepicondyloulnaris, extensor longus alulae, flexor digitorum profundus, and ulnometacarpalis dorsalis. Scale bar = 40 mm.

### M. **pronator superficialis** (**Figs 5**, **14**, **15**)

The tendinous origin of this muscle lies on the proximal ventral epicondyle of the humerus. The proximalmost of several pits found on the ventral epicondyle is associated with this muscle’s tendon of origin. The fleshy belly of this muscle covers much of the surface of M. pronator profundus, and contacts the surface of the ventral radial shaft in a small, teardrop-shaped area demarcated entirely by intermuscular lines, as a small tendon.

### M. **pronator profundus (****Figs 5**, **14**, **15**)

This muscle’s tendinous origin is located further distally on the ventral epicondyle of the humerus than that of M. pronator superficialis, and is associated with a relatively deep circular pit on the bone. It has a fleshy insertion on the proximal ventral radial shaft, extending for approximately one fifth the length of the radius. The insertion extends slightly further distally than that of M. pronator superficialis, and is demarcated by intermuscular lines.

### M. **entepicondyloulnaris (****Figs 13**, **15**)

M. entepicondyloulnaris and M. pronator profundus are closely associated, and in many places are difficult to distinguish from one another in our CT dataset. The origin is by a common tendon with M. pronator profundus. The entirety of the fleshy belly of M. entepicondyloulnaris lies deep to that of M. pronator superficialis and exists as an extremely thin layer, almost completely fused to M. pronator profundus. The insertion is fleshy on the proximal ventral ulnar shaft, caudal to that of M. brachialis. No osteological correlates were found in the area of insertion. This muscle is notable for being found only in palaeognaths and galloanserans (Hudson and Lanzillotti 1964; Hudson et al. 1972; Vanden Berge and Zweers 1993; Livezey and Zusi 2006; Suzuki et al. 2014; Widrig et al. 2023), and as such is sometimes referred to as the “gallinaceous muscle” in older literature (George and Berger 1966).

### M. **flexor digitorum superficialis (****Figs 5**, **12**, **15**)

The tendinous origin of this muscle is located on the distal ventral epicondyle. The tendon of origin is extremely long, and does not expand into a fleshy belly until it reaches approximately the distal fifth of the forearm. The tendon of origin lies superficial to M. flexor carpi ulnaris throughout its length. In our CT data, the tendon is no longer visible distal to the radiale. Upon dissection of the specimen, we observed the tendon to cross the wrist on its ventral side, looping around the ulnare. It then passes dorsally to reach the tendon of M. flexor digitorum profundus, with which it is closely associated as it traverses the ventrocranial side of the major metacarpal to insert on the proximalmost cranial side of phalanx I of the major digit. A small tubercle is associated with the insertion. This contradicts Livezey and Zusi (2006), who coded this muscle as inserting on the distal phalanx of the major digit in *Chauna* and *Anseranas*, and on the proximal phalanx of the major digit in *Anas* and *Anser*.

### M. **flexor digitorum profundus (****Figs 12**, **13**, **15**)

The proximal origin of this muscle abuts the distalmost area of insertion of M. entepicondyloulnaris on the proximal ventral ulnar shaft. The origin of M. flexor digitorum profundus is fleshy, and stretches across approximately the middle two-fourths of the ulna, leaving intermuscular lines on the cranioventral ulnar shaft. At three fourths the length of the ulna it becomes tendinous. The tendon of insertion passes superficially to the belly of M. ulnometacarpalis ventralis and across the wrist cranial to the ulnare. It turns about the pisiform process to extend down the length of the cranioventral major metacarpal along with the tendon of M. flexor digitorum superficialis. It crosses over the dorsocranial end of phalanx 1 of the major digit to insert on the proximal dorsal portion of the second phalanx. No osteological correlates are associated with the insertion. This again contradicts Livezey and Zusi (2006), who coded this muscle as inserting on the proximal phalanx of the major digit in *Chauna* and *Anseranas*, and on the distal phalanx of the major digit in *Anas* and *Anser*.

### M. **flexor carpi ulnaris (****Figs 5**, **12**, **15**)

This is one of the largest antebrachial muscles. It takes a relatively wide origin wrapping around the distal face of the ventral epicondyle (the flexor process), which has a rugose surface. The belly of the muscle runs parallel to the caudal surface of the ulna and is fleshy for most of its length. We did not observe any connections between this muscle and the bases of the secondary remiges in the CT dataset, unlike in the tinamou digitally dissected by Widrig et al. (2023) in which they were clearly visible in the CT data. We observed their presence upon dissecting the antebrachium and found that they were encased in thick fibrous tissue, which likely prevented the uptake of stain. M. flexor carpi ulnaris inserts via a thick tendon on the muscular process of the proximal ulnare.

### M. **ulnometacarpalis ventralis (****Figs 12**, **13**, **15**)

The distal cranial ulnar shaft is the site of this muscle’s fleshy origin, which is bounded by intermuscular lines. It crosses the wrist as a thick tendon through a clearly defined notch on the ventral side of the radiale identified by Mayr (2014) and inserts on the proximal cranial carpometacarpus, where it does not leave any osteological correlates.

### M. **extensor carpi radialis** (**Figs 5**, **10**, **12**, **15**, **16**)

This large muscle contains two distinct bellies. The more proximal belly originates from the proximal dorsal epicondyle but has an additional origin from the dorsal humerus at approximately two thirds of the way down the humeral shaft. A pit on the proximal dorsal epicondyle is associated with the main origin, but no correlate exists for the origin on the humeral shaft. The more distal belly has a long tendon of origin from the proximal dorsal epicondyle immediately adjacent to the tendon for the first belly, which widens into a fleshy belly at approximately one fourth of the way down the radial shaft. At approximately halfway down the length of the radius, the bellies fuse, and the muscle thins into a very long tendon. The tendon passes over the distal end of the radius to insert on the proximodorsal base of the proximal bony spur of the carpometacarpus, equivalent to the position of the extensor process in taxa that lack such spurs. A long transverse scar is associated with this muscle’s insertion and that of Mm. propatagialis and extensor longus alulae on either side.

**Fig. 16:**
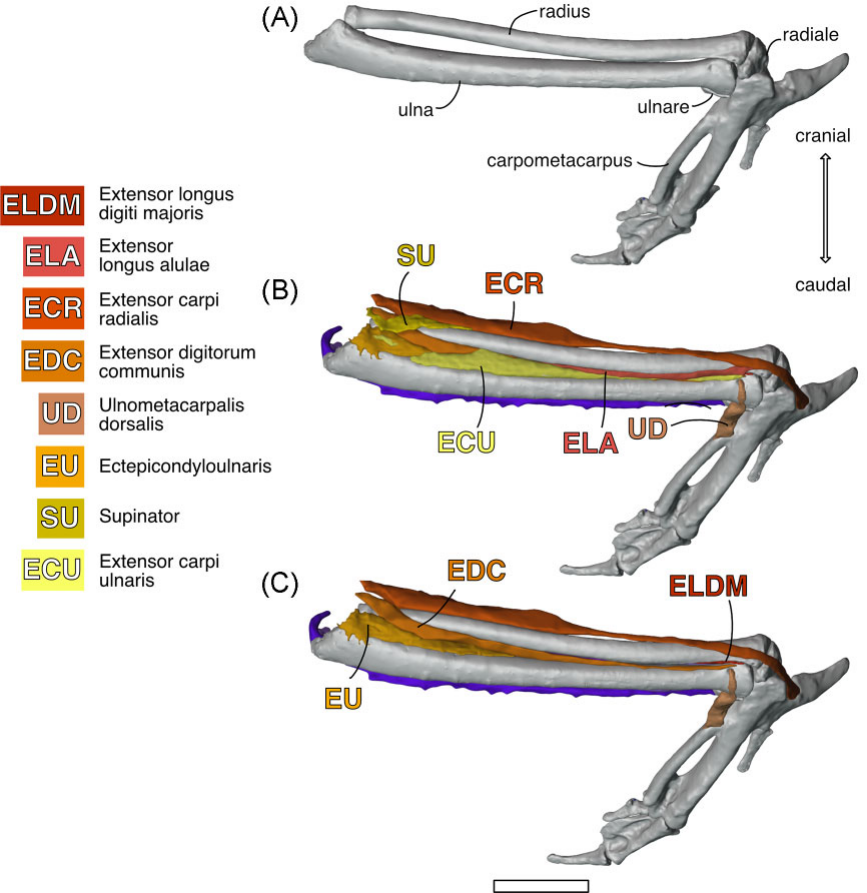
Digitally segmented forearm of *Chauna torquata* in dorsocaudal view. A: Right radius, ulna, radiale, ulnare, carpometacarpus, and phalanges. B: same osteology as A, with right Mm. supinator, extensor carpi ulnaris, extensor carpi radialis, and ulnometacarpalis dorsalis. C: same osteology as A, with M. extensor carpi ulnaris removed to display the deeper muscles of the forearm: Mm. ectepicondyloulnaris, extensor digitorum communis, and extensor longus digiti majoris. Scale bar = 40 mm.

### M. **supinator** (**Figs 5**, **14**, **16**)

This relatively small muscle has a tendinous origin on the proximal cranial face of the dorsal epicondyle of the humerus, just proximal to that of M. extensor digitorum communis. The insertion is fleshy on the proximal radial shaft, extending slightly less than one fifth the length of the bone. No osteological correlates are associated with its origin, but its insertion is on a very clear rugose area on the surface of the radius bounded by intermuscular lines.

### M. **extensor digitorum communis (****Figs 5**, **12**, **16**)

The origin is by a relatively thick tendon to the proximal cranial side of the dorsal epicondyle that is closely associated with but separate from the M. supinator tendon of origin. There is no corresponding osteological correlate for the insertion. The fleshy belly of the muscle extends approximately one third the length of the radius, and lies deep to M. extensor carpi ulnaris for much of its length. The tendon of insertion crosses the wrist in close association with those of Mm. extensor carpi ulnaris and extensor longus digiti majoris, but cannot be observed in the CT dataset distal to this point, likely due to thick overlying connective tissue that prevented the uptake of stain. We confirmed that the tendon of insertion splits, with one branch inserting on the caudal edge of the alular phalanx and the other passing through the tendinal groove on the dorsal carpometacarpus to insert on the proximalmost cranial edge of phalanx I of the major digit, where it leaves a tiny impression.

### M. **ectepicondyloulnaris (****Figs 5**, **13**, **16**)

This muscle is alternatively called M. anconeus, especially in older literature. The flat, wide tendon of origin is not visible in our CT dataset, but we confirmed via dissection that it attaches to the apex of the dorsal epicondyle, and does not have any associated osteological correlates. As it crosses the elbow, the tendon expands to form the main belly of the muscle. The insertion is fleshy on the proximal cranial ulna, extending approximately halfway down the shaft, and intermuscular lines associated with this muscle can be seen on the cranioventral ulnar shaft proximal to those of M. flexor digitorum profundus. The belly of M. ectepicondyloulnaris contacts the radius proximally.

### M. **extensor carpi ulnaris (****Figs 5**, **12**, **16**)

The tendon of origin is not visible in our CT dataset, but we confirmed that its origin is from the apex of the dorsal epicondyle, just proximal to that of M. ectepicondyloulnaris. There is no associated osteological correlate. The fleshy belly of the muscle lies superficial to the other muscles of the dorsal forearm. It narrows to a tendon at approximately two thirds down the length of the forearm, and is closely associated with the tendons of Mm. extensor longus digiti majoris and extensor digitorum communis, but can no longer be observed distal to the wrist in our CT data. We confirmed that it has a typical insertion on a raised scar on the dorsal carpometacarpus just proximal to the interosseous space.

### M. **extensor longus alulae (****Figs 12****-****15**)

This muscle has a dual fleshy origin from the radius and ulna on each side of the interosseous space, and as such is one of the deepest muscles of the forearm. The radial head is much larger than the ulnar head, with the origin extending along nearly the entire length of the radial shaft. There is a distinct intermuscular line on the radius separating Mm. extensor longus alulae and pronator profundus, but no correlates were found on the ulna. The two heads merge into a single belly at about one fifth the length of the radius. The tendon of insertion passes cranially to join the bundle of tendons crossing the wrist with that of M. extensor carpi radialis, and can no longer be distinguished as a separate entity distal to this point in the CT dataset. We confirmed via dissection that it inserts just dorsal to M. extensor carpi radialis on the extensor process.

### M. **extensor longus digiti majoris (****Figs 12**, **14**, **16**)

This thin muscle originates along the caudoventral margin of the distal third of the radius, and is closely associated with M. extensor longus alulae. The distal portion of the origin leaves associated intermuscular lines. It narrows to a tendon and cannot be observed distal to the wrist in our CT data. We confirmed that it crosses the wrist with the tendons of Mm. extensor digitorum communis and extensor carpi ulnarisinserts on the proximalmost dorsocranial edge of phalanx II, where it leaves a tiny rugose tubercle.

### M. **ulnometacarpalis dorsalis (****Figs 12**, **13**, **15****-****17**)

The tendon of origin for this muscle stretches across the caudal surface of the distal head of the ulna, where it leaves a clear impression on the bone surrounded by intermuscular lines. The muscle belly crosses the wrist and inserts fleshily on the proximalmost portion of the minor metacarpal, where it leaves no osteological correlate.

**Fig. 17:**
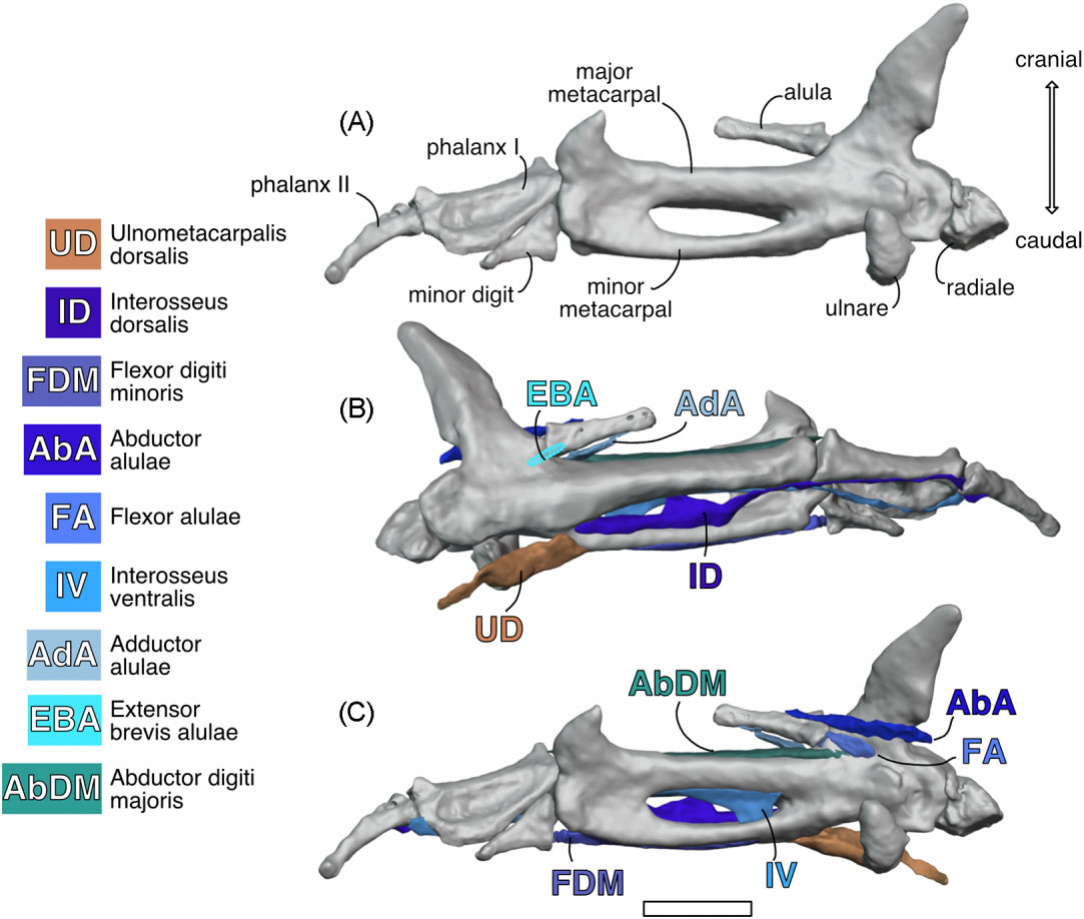
Digitally segmented muscles of the manus of *Chauna torquata*. A: osteology of the manus (right radiale, ulnare, carpometacarpus, and phalanges) in original articulation in ventral view. B: same osteology as A, with Mm. ulnometacarpalis dorsalis, interosseus dorsalis, adductor alulae, and extensor brevis alulae, in dorsal view. M. extensor brevis alulae could not be visualized in our CT scans; its location was verified by dissection and is shown here with dashed lines. C: same osteology as A, with Mm. abductor alulae, flexor alulae, abductor digiti majoris, interosseus ventralis, and flexor digiti minoris, in ventral view. Scale bar = 25 mm.

### M. **abductor alulae (****Figs 12**, **17**)

The fleshy origin of this muscle is located on the tendon of insertion of the M. extensor carpi radialis. It extends across to the alular phalanx, inserting on its proximal cranioventral edge. No osteological correlates could be found for this muscle.

### M. **flexor alulae (****Figs 12**, **17**)

The fleshy origin and belly of the muscle are located on the ventral alular metacarpal, between the pisiform process and the proximal spur. The area of origin is smoother than the bone surrounding it. The muscle inserts via a tiny tendon on a tubercle on the proximalmost edge of the ventral alular phalanx.

### M. **adductor alulae (****Figs 12**, **17**)

The origin is on the cranioventral edge of the proximal major metacarpal, directly abutting the origin of M. abductor digit majoris. The insertion is about halfway down the length of the alular phalanx, on its caudal edge. There are no associated osteological correlates.

### M. **extensor brevis alulae (****Figs 12**, **17**)

This muscle is not visible in our CT dataset. Upon dissecting the stained specimen, we found that this minute muscle has a fleshy origin on the dorsal alular metacarpal, and inserts via a tiny tendon on the dorsocaudal corner of the proximalmost alular phalanx. We found fibrous connective tissue overlying this muscle, which likely prevented the uptake of stain. No osteological correlates could be found for the origin, but there is a tiny tubercle associated with the insertion.

### M. **abductor digiti majoris (****Figs 12**, **17**)

The fleshy origin of this muscle extends along nearly the entire cranial surface of the major metacarpal, and is associated with intermuscular lines. It is overlain by the insertion tendon of M. flexor digiti profundus. The tendon of insertion can no longer be visualized in our CT dataset distal to the major metacarpus. We found the insertion site to be on a rugose tubercle on the proximalmost cranial side of phalanx I of the major digit.

### M. **interosseus dorsalis (****Figs 12**, **17**)

The fleshy origin is on the caudal side of the interosseal membrane and the cranial side of the minor metacarpal. A small additional slip joins the main belly from the approximate midpoint of the major metacarpal facing the interosseous space. Distal to the interosseous space, it thins out into a tendon that inserts on the proximalmost caudal side of the second phalanx of the major digit. There are no associated osteological correlates with the origin and insertion of this muscle, but a clear tendinal sulcus is present running along the craniodorsal side of phalanx I of the major digit.

### M. **interosseus ventralis (****Figs 12**, **17**)

Most of the fleshy origin lies along the caudal major metacarpal facing the interosseous space, with an additional slip originating on the cranial proximal minor metacarpal. An intermuscular line is present on the ventral side of the interosseous space. The tendon of insertion exits the interosseous space on the dorsal side of the carpometacarpus, and extends along a groove on the caudal edge of the first phalanx of the major digit to insert on a small tubercle on the proximalmost caudal side of phalanx II.

### M. **flexor digiti minoris (****Figs 12**, **17**)

This muscle, long and thin, takes its fleshy origin from the caudal edge of the distal two thirds of the minor metacarpal, where it leaves intermuscular lines. The insertion is on a tubercle at the proximalmost caudal end of the minor digit.

## Discussion

### Comparison with Cygnus

Here we compare the anatomy of *Chauna* from this study to the description of the Whooper Swan (*Cygnus cygnus*) from Matsuoka and Hasegawa (2007), one of the only other available publications concerning the wing anatomy of an anseriform. The authors treated muscles associated with the sternum, coracoid, furcula, scapula, and humerus only, leaving out the antebrachium and manus. As one of the heaviest volant birds, *Cygnus cygnus* is even more massive than *Chauna*, with the specimen dissected by the authors weighing 7250 grams. Unlike the soaring flight of screamers, flight in swans is characterized by continuous wingbeats that may be interspersed with brief periods of gliding. Taking off from water after a running start, swans are powerful long-distance migratory fliers that can maintain airspeeds of 100 kph (Matsui et al. 1980; Mlodinow et al. 2025).

**Fig. 18:**
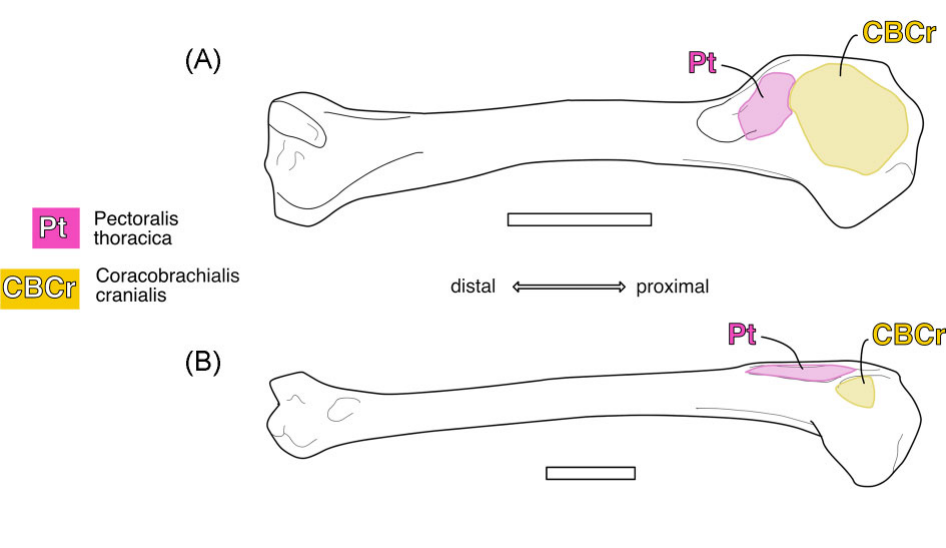
Comparison of insertions on the proximal humeri of *Chauna* and *Cygnus*. A: Right humerus of *Chauna torquata* in cranial view, showing insertion areas of Mm. pectoralis thoracica and coracobrachialis cranialis. B: Right humerus of *Cygnus cygnus* in cranial view, showing insertion areas of Mm. pectoralis thoracica and coracobrachialis cranialis. Scale bars = 40 mm. *Cygnus* illustration based on Matsuoka and Hasegawa (2007) and osteological specimen USNM 430049.

While broadly similar, differences between the pectoral and shoulder muscles of *Cygnus* and *Chauna* could be reflective of their differences in flight style. The origin of M. coracobrachialis caudalis extends onto the sternum in *Cygnus*, but not in *Chauna* (Fig. 7). In *Chauna*, the same region is instead occupied by M. pectoralis (Fig. 6). The insertion of M. pectoralis is displaced distally in *Chauna* relative to *Cygnus*, with most of the proximocranial deltopectoral crest occupied by M. coracobrachialis cranialis in *Chauna* (Figs 5, 18). In *Cygnus*, the latter muscle is restricted to a smaller coracobrachial impression. It is possible that the more expansive insertion of M. coracobrachialis cranialis in *Chauna* is simply due to the distal displacement of M. pectoralis, and not an increased importance of this muscle. The more proximal insertion of M. pectoralis relative to total humeral length in *Cygnus* compared to *Chauna* may be related to the former’s tendency to utilize rapid continuous wingbeats, as a proximal insertion on the humerus achieves greater wingbeat speed at the cost of leverage (Houde 1988; Serrano and Chiappe 2017). In addition, a more proximal insertion may contribute to a craniolateral orientation of M. pectoralis lines of muscle action, leading a greater amount of force produced by M. pectoralis to contribute to wing retraction. This aids in counteracting the aerodynamic forces produced by vigorous wing flapping.

Meanwhile, soaring birds must counteract upward aerodynamic forces acting upon the wing surface, which favors laterally oriented lines of muscle action for M. pectoralis. In this case, a distal insertion of M. pectoralis, like that seen in *Chauna*, may be advantageous (Akeda and Fujiwara 2026).

Other minor differences in shoulder musculature include that M. deltoideus major has two heads in *Cygnus* but only one in *Chauna*, M. scapulotriceps has an additional origin on the furcula in *Cygnus* that is absent in *Chauna*, and that M. subscapularis has a pars interna and externa in *Chauna*, but pars externa is absent in *Cygnus*. The loss of M. subscapularis pars externa has not been observed in other neognaths, with the only other published records of the absence of this subdivision occurring in flightless palaeognaths (Beddard 1898; McGowan 1982; Jasinoski et al. 2006; Maxwell and Larsson 2007; Widrig et al. 2023). M. scapulohumeralis cranialis is present in *Cygnus*, while it is present but clearly vestigial in *Chauna*. Given its appearance in *Cygnus* and in all 31 galliform genera examined by Hudson and Lanzillotti (1964), the extreme reduction of this muscle in *Chauna* appears to be apomorphic. Unlike the loss of M. subscapularis pars externa in *Cygnus*, this muscle has been lost multiple times independently within Neognathae (George and Berger 1966; Vanden Berge 1970; McKitrick 1991; Razmadze et al. 2018).

### Comparison with *Camptorhynchus*

A report on the musculature of the antebrachium and manus of the extinct Labrador Duck (*Camptorhynchus labradorius*) by Zusi and Bentz (1978) based on a study skin with preserved musculature (USNM 1972) is the only publication on anseriform distal wing elements available. The last reported sighting of this seaduck (Tribe Mergini, subfamily Anatinae) occurred in 1878 in Elmira, New York (Chilton 2020). Very little information pertaining to the life history of *Camptorhynchus* was recorded prior to its extinction, but surviving accounts suggest that it was a strong, swift flier (Audubon 1843; Chilton 2020). Its living sister taxon is the Steller’s Eider (*Polysticta stelleri*) (Buckner et al. 2018), whose flight is described as similar to scaups with rapid continuous wingbeats and the ability to take off quickly from water (Delacour 1959; Fredrickson 2020).

We found many differences in the relative lengths of radial and ulnar insertions between *Chauna* and *Camptorhynchus*, which may be related to *Chauna* possessing relatively longer wings due to its preference for soaring flight. More proximally located insertions serve to reduce the distal inertia of an elongated wing (Houde 1988), and we observed many examples of this trend in *Chauna*. M. supinator has a significantly longer insertion in *Camptorhynchus*, occupying the proximal half of the radius. The insertion of M. pronator superficialis extends along the proximal half of the radius in *Camptorhynchus*, while this muscle has a tiny insertion area in *Chauna* (Fig. 14). M. pronator profundus likewise inserts on a greater extent of the radius in *Camptorhynchus* than in *Chauna* (Fig. 14). In *Camptorhynchus*, M. ulnometacarpalis ventralis has a larger origin that extends over the distal half of the ulna, and M. ectepicondyloulnaris extends over the proximal two thirds of the ulna in *Camptorhynchus* but only over the proximal third in *Chauna* (Fig. 13).

Both *Camptorhynchus* and *Chauna* have an M. extensor carpi radialis that is divided into two bellies, with the main belly in each species originating from the dorsal epicondyle just proximal to the origin of M. supinator (Fig. 5). Unlike *Chauna*, in which there is a secondary origin of the main belly from the humeral shaft, in *Camptorhynchus* the origin is restricted to the dorsal epicondyle. In both species, M. entepicondyloulnaris shares its tendon of origin with M. pronator profundus. The muscle is apparently much more developed in *Camptorhynchus*, as Zusi and Bentz state that it is “clearly present,” and was found in the other ducks dissected for that study.

We also observed differences in the more distal antebrachial and manual musculature. The origin of M. extensor longus digiti majoris occupies most of the caudal radius in the duck, but just the distal quarter of the caudal radius of *Chauna* (Fig. 14). In the duck, M. flexor digitorum superficialis attaches to the proximal end of phalanx I of the major digit, but the tendon continues and inserts onto the cranial surface of proximal phalanx II, whereas it terminates at phalanx I in *Chauna* (Fig. 12). M. flexor digitorum profundus inserts on the third phalanx of the major digit in *Camptorhynchus*, whereas *Chauna* lacks this terminal digit. The presence of phalanx III in the duck also affects the insertion of M. interosseus dorsalis, which inserts on the third phalanx in *Camptorhynchus*.

### Anhimid osteological correlates and flight muscle form-function relationship

After finding that only 29% of wing muscle attachments on bones in the North Island brown kiwi (*Apteryx mantelli*) can be identified from specific osteological features, McGowan (1982) cautioned against utilizing osteological features to reconstruct myology in extinct birds. By contrast, we found that 60% of muscle origins and 80% of muscle insertions leave recognizable osteological correlates in *Chauna*. Our interpretation of this result is that the kiwi may simply represent an extreme case due to its reduced wings, and that osteological correlates remain a valid tool for reconstructing the musculature of extinct birds. The distal extent of intermuscular lines correlated with antebrachial muscles could be another way to assess flight style in extinct taxa in addition to skeletal metrics such as sternum shape (Lowi-Merri et al. 2021; Lowi-Merri et al. 2023; Widrig et al. 2025). Our finding of a relatively high fidelity between flight-related muscles and osteological correlates of their attachment means that this dataset could indeed prove useful in reconstructing wing myology and making inferences regarding flight style in early Anseriformes such as presbyornithids, and looking further stemward, could be used in tandem with information from volant palaeognaths for reconstructions both in early crown group birds and birds just outside the crown group.

An additional group which could benefit from reconstructions based on the present dataset are the pelagornithids, a clade of giant marine “pseudo-toothed” birds that went extinct in the Pliocene (Boessenecker and Smith 2011). The largest species, *Pelagornis sandersi*, had an estimated wingspan of up to 6.4 meters, making it one of the largest flying birds of all time (Ksepka 2014). Unsurprisingly, their extreme size relative to extant marine birds has generated interest in their feeding ecology (Hellyer-Price et al. 2026) and flight performance within a presumed soaring flight style (Goto et al. 2022). Their phylogenetic affinities have been controversial (Mayr 2011), having once been placed in a now paraphyletic “pelecaniform” clade (Olson 1985) before more recently being found as the sister group to Anseriformes (Bourdon 2005). Their anseriform-like palate may actually represent a neornithine plesiomorphy, which could potentially place them further stemward among Neornithes, or even outside the avian crown group altogether (Mayr 2011; Mayr et al. 2021; Benito et al. 2022). Regardless of whether they belong within total group Anseriformes or are further stemward on the avian tree of life, information from screamers, as the earliest-diverging extant soaring birds, can be useful for reconstructing their flight musculature.

Our finding that the wing musculature of *Chauna* is similar to that found in other soaring birds is in keeping with findings of other authors regarding anhimid morphology. As with most other thermal soarers, screamers have wings with a low aspect ratio and relatively low wing loading, while ducks have an intermediate aspect ratio with relatively high loading (Rayner 1988). Additionally, screamers possess a subpectoral diverticulum, a projection of the interclavicular air sac which increases the moment arm of the cranial pectoralis muscle that has evolved at least seven times independently in soaring lineages (DeMay 1940; Schachner et al. 2024). Our finding of a somewhat distal insertion of M. pectoralis suggests specialization for slower, more powerful wingbeats (Houde 1988; Rayner 1988; Serrano and Chiappe 2017), as opposed to a more proximal insertion which allows for greater wingbeat speed at the cost of mechanical power found in other anseriforms. A similar distal insertion of M. pectoralis is found in other thermal soaring specialists such as cathartids, which also share the trait of having antebrachial muscles with insertions that do not extend as far distally, thus reducing the distal inertia of the wing (Fisher 1946). Burst flying birds are adapted for even more rapid wingbeats than those seen in ducks, geese, and swans, and possess an even more proximally located insertion of M. pectoralis, insertions of antebrachial muscles that cover most of the forearm, and a very large M. supracoracoideus relative to M. pectoralis for a rapid upstroke (Hudson and Lanzillotti 1964; Hudson et al. 1972; Rayner 1988; Suzuki et al. 2014; Widrig et al. 2023). Screamers, cathartids, and other soarers all appear to exist on one end of a musculoskeletal continuum, with burst fliers such as phasianids and tinamids occupying the other, and Anseres falling somewhere in the middle.

The latest molecular divergence dates suggest that anhimids diverged from the remaining Anseriformes approximately 49 million years ago (Stiller et al. 2024), leaving a huge span of time for musculoskeletal differences to accumulate. M. extensor carpi radialis has a somewhat unusual morphology in *Chauna*. A secondary origin on the humeral shaft similar to that seen in *Chauna* does not appear to be present in other anseriforms but has been reported in *Corvus* (Hudson and Lanzillotti 1955). It is not clear if there is any functional significance to this. In *Corvus* there is a large tubercle associated with the humeral shaft origin, but none is present in *Chauna*.

One notable difference from other anseriforms is the near vestigiality of M. entepicondyloulnaris in *Chauna*. This muscle is present in ducks (Zusi and Bentz 1978) and all galliforms surveyed by Hudson and Lanzillotti (1964), while in *Chauna* it is reduced to a very small bundle of fibers branching off from M. pronator profundus. This muscle, when present, functions to depress the distal end of the forearm (Hudson and Lanzillotti 1955). This is somewhat redundant with the function of Mm. pronator superficialis and pronator profundus, which rather than serving as antebrachial pronators in a strict sense instead serve to flex and depress the forearm (Fisher 1946; Owre 1967; McGowan 1986). Given that M. entepicondyloulnaris is absent in all Neoaves, it is clearly not necessary for complex flight, and there apparently was not strong enough selective pressure to maintain this muscle as a well-developed belly in *Chauna*. Possibly it is reduced in anhimids because they typically soar while holding the wings in a steady posture, and M. entepicondyloulnaris is largely redundant given the presence of two functional pronators. This raises the further possibility that the common ancestor of Neoaves flew in a similar way and thereby lost this muscle, but given the sparse fossil record of early neoavians, this is highly speculative.

M. scapulohumeralis cranialis is notably reduced in *Chauna*, and its variable appearance across disparate branches of the avian family tree suggest it has been lost many times independently (George and Berger 1966; Vanden Berge 1970; McKitrick 1991; Razmadze et al. 2018). Meyers (1992) posits that although this muscle’s main function appears to be to adduct and retract the humerus, in actuality its function is more likely postural due to its tonic fibers and small size. It is confirmed to be present in Tinamidae (Hudson et al. 1972; Suzuki et al. 2014; Widrig et al. 2023), Galliformes (Hudson and Lanzillotti 1964), *Cygnus* (Matsuoka and Hasegawa 2007), Cuculidae, (Berger 1954), Musophagidae (Berger 1960), *Grus* (Fisher and Goodman 1955), *Gallirallus*, (McGowan 1986), *Larus* and *Uria* (Hudson et al. 1969; Watanabe et al. 2021), *Pelecanoides* (McKitrick 1991; Watanabe et al. 2021), *Nycticorax* (Vanden Berge 1970), *Coragyps* and *Gymnogyps* (Fisher 1946), *Falco* (Meyers 1992), *Tyrannus* (McKitrick 1985), *Corvus* (Hudson and Lanzillotti 1955), and *Agelaius* (George and Berger 1966), but is absent in *Columba* (George and Berger 1966) and *Psittacus* (Razmadze et al. 2018). Notably, it is variably present in *Gavia immer* (McKitrick 1991), and within the genus *Phoenicopterus*, it is present in *P. chilensis* and *P. jamesi*, but not in *P. ruber* (Vanden Berge 1970). Given its clear presence in *Cygnus* and in tinamids, its vestigiality in *Chauna* likely represents an apomorphic feature, and we conclude that this muscle was present in both the common ancestor of Galloanserae and Neornithes.

### Comments on methodology

Our *Chauna torquata* specimen approached the upper limit of object size that could be imaged by the GE phoenix v|tome|x m microCT scanner. Accordingly, staging the specimen such that it was entirely within the X-ray beamline was difficult. Another challenge posed by the size of the specimen was that due to the relatively slow rate of iodine perfusion deep into the interior of the specimen, we ran the risk of overstaining the superficial musculature of the bird while understaining the deeper tissues. Our test scan after 42 days of staining revealed that while the musculature of most of the brachium, antebrachium, and manus was nearly fully stained and could be visualized, the musculature within the body cavity had not absorbed sufficient iodine to be differentiated, particularly Mm. pectoralis and supracoracoideus (Fig. 3). We attempted to mitigate this by injecting additional iodine stain into the right side of the specimen, but the final scan revealed that the amount of iodine injected was insufficient to have any visible effect. Despite the muscles of the pectoral region not being as clearly differentiated as the superficial musculature by the time of the final scan, we deemed it sufficient to carry on with the project as we wanted to avoid overstaining the exterior of the specimen (Gray et al. 2024).

Early et al. (2020) reported understaining of the viscera in their specimen stained in 70% I_2_, despite utilizing a significantly smaller bird species (the House Sparrow *Passer domesticus*). Gray et al. (2024) utilized daily injections of iodine to stain a turtle specimen and reported positive results compared to a turtle that was simply submerged in stain without injections, as the carapace obstructed diffusion into organs and internal tissues. By contrast, Kolmann et al. (2023) report that injection did not speed up their stain uptake times in fishes. Our recommendation is for future researchers to attempt performing injections into the body cavity with some regularity for larger specimens, and we stress the need for further investigation of appropriate methods for staining large specimens. On a more positive note, our destaining of the specimen largely appears to have been successful in removing brown iodine stain, and we did not observe the demineralization leading to flexible bones observed by Early et al. (2020) in their specimen stained in Lugol’s iodine. This supports the finding that ethanol-based stains cause less demineralization, but could also be due in part to the much greater size of our specimen and less exposure per gram of body mass to the stain, considering that our specimen was not as completely stained as those of Early et al. (2020).

The total time necessary for adequate staining varies with taxonomic group in addition to body size. Reptiles and amphibians have been observed to stain faster than mammals and fishes of similar body size (Gray et al. 2025). Kolmann et al. (2023) note that the rate difference at which large and small fish uptake stain is not statistically significant, meanwhile Callahan et al. (2021) report optimal staining times increase linearly with body size for snakes. Because the large scale study of Gray et al. (2025) did not include birds, it is more difficult to assess where birds fall along this continuum to assist future researchers in estimating the total time needed for staining. Although there are several examples of contrast-enhanced CT studies of birds in the published literature, direct comparisons between them are difficult because of the different methodologies used. For example, some use whole specimens (Early et al. 2020; Widrig et al. 2023), while others utilize partially dissected and or skinned birds (Bribiesca-Contreras and Sellers 2017; Sullivan et al. 2019). The variety of stains used (iodine-buffered formalin, Lugol’s solution, or elemental iodine in either ethanol or water) also adds to the difficulty of cross comparison between studies, highlighting the need for additional methodological studies concerning the staining and scanning of birds.

A noted drawback of diceCT is that tendinous muscle attachments can be difficult to visualize (Bribiesca-Contreras and Sellers 2017; Collings and Richards 2019; Widrig et al. 2023), and may require the partial dissection of the original specimen to confirm. We found this to be the case in several instances for this study, and we caution researchers looking to create three-dimensional anatomical atlases of musculature for other species of the likelihood that they will have to fall back on traditional dissection for some muscles. Thus, this method cannot be considered minimally destructive to specimens, and researchers and collection managers should be aware of this when processing specimen loan requests. Additionally, while myological descriptions using digital dissections from CT data are valuable for the sharable 3D datasets that they generate, traditional dissection is much more efficient in terms of both time and resources in obtaining information pertaining to muscle sizes, positions, and origins and insertions. Despite growing interest in the use of artificial intelligence to speed up image analysis, it is not yet applicable to this context due to each muscle having the same or very similar grey values, which inhibits auto-segmentation.

Dissection is more efficient in terms of time spent obtaining this basic information, but the generation of 3D datasets via diceCT is of particular utility for downstream application to biomechanical modelling. In addition to data on muscle origins and insertions, these datasets can also preserve muscle architecture information, such as fascicle length and orientation and muscle volume (Sullivan et al. 2019), which are crucial for biomechanical models of musculoskeletal function (Demuth et al. 2022). Further applications exist for muscle reconstruction and biomechanical modeling in extinct species. Reasonable assumptions regarding muscle origins and insertions can be made via observation of osteological correlates and extant phylogenetic bracketing. Following the methods of Demuth et al. (2022), volumetric reconstruction of 3D muscle bellies can then be performed, which produces lines of action that can be used in simulation software. Such reconstructions require model validation using related extant taxa, which is where 3D datasets such as that of the present study are extremely valuable. Given that they are archivable and sharable, 3D datasets from extant species will significantly ease comparison with the generated models than would otherwise be possible with data from traditional dissection alone. If volumetric reconstruction of muscles in the extant validation taxon produce a dataset that is minimally different from that generated from diceCT, one can have greater confidence in the reconstruction of the extinct taxon (Demuth et al. 2022). With this goal in mind, the time and effort required to generate diceCT datasets is well justified. The present dataset now represents one of the few available for the avian wing in three dimensions. Given the amount of interest in the evolution of avian flight, we believe that this dataset will provide a valuable resource to those interested in modeling the musculoskeletal system of extinct avialans.

## Conclusions

Our findings showcase the benefits and drawbacks of using diceCT for myological studies and highlight the need for further investigation into best practices for staining larger specimens, particularly how to ensure viscera and deep tissues are stained without overstaining the extremities. Despite these challenges, diceCT is an exciting frontier of data collection with powerful downstream applications, and our work here provides useful 3D data for an understudied clade. By providing a three-dimensional myological atlas of a bird from an ancient lineage, we shed light on the diversity of anatomy within the extant bird radiation, as well as provide a basis for future paleontological work on enigmatic extinct birds such as pelagornithids. Our hope is that this dataset, in combination with previous publications on avian wing anatomy, will provide a foundation for further study of the evolution of the musculoskeletal system that powers avian flight, one of the most extraordinary adaptations in the animal kingdom.

## Data Availability

MorphoSource Project ID: 000839482. Zenodo DOI: 10.5281/zenodo.18985236

## Supplementary data

Scan data and meshes produced by this study are archived on MorphoSource. A full three-dimensional model constructed in Blender is available on Zenodo.
